# Bayesian Multiple Emitter Fitting using Reversible Jump Markov Chain Monte Carlo

**DOI:** 10.1038/s41598-019-50232-x

**Published:** 2019-09-24

**Authors:** Mohamadreza Fazel, Michael J. Wester, Hanieh Mazloom-Farsibaf, Marjolein B. M. Meddens, Alexandra S. Eklund, Thomas Schlichthaerle, Florian Schueder, Ralf Jungmann, Keith A. Lidke

**Affiliations:** 1 Department of Physics and Astronomy, University of New Mexico, Albuquerque, New Mexico USA; 2 Department of Mathematics and Statistics, University of New Mexico, Albuquerque, New Mexico USA; 3 Department of Physics and Center for Nanoscience, Ludwig Maximilian University, Munich, Germany; 4 Max Planck Institute of Biochemistry, Martinsried, Germany

**Keywords:** Super-resolution microscopy, Single-molecule biophysics

## Abstract

In single molecule localization-based super-resolution imaging, high labeling density or the desire for greater data collection speed can lead to clusters of overlapping emitter images in the raw super-resolution image data. We describe a Bayesian inference approach to multiple-emitter fitting that uses Reversible Jump Markov Chain Monte Carlo to identify and localize the emitters in dense regions of data. This formalism can take advantage of any prior information, such as emitter intensity and density. The output is both a posterior probability distribution of emitter locations that includes uncertainty in the number of emitters and the background structure, and a set of coordinates and uncertainties from the most probable model.

## Introduction

In single molecule localization microscopy (SMLM) super-resolution approaches^1–4^, a sparse subset of single fluorescent emitters that label the target structure is activated and the position of each isolated emitter is found with a precision much better than the diffraction limit. Accumulation of enough label positions allows the reconstruction of images with high spatial resolution^5^. Dense images with overlapping emitters can be either unavoidable due to densely labeled structures, or desired to shorten data collection time. However, improper analysis of this data can lead to artifacts, such as a contrast inversion in the super-resolution image (dense areas appear sparse)^6^. One way to ameliorate this issue is to use multiple-emitter fitting approaches^6–12^, which allow modeling and/or fitting of multiple overlapping emitters. Several multiple-emitter fitting methods have been reported including approaches based on maximum likelihood^6^, deconvolution with L1 norm constraints^9,10^, PSF radial symmetry and intermittency^11^, using a Bayesian approach to integrate over all possible positions and blinking events of emitters^7^, and deep learning^13,14^.

In this work, we describe a BAyesian Multiple-emitter Fitting (BAMF) analysis that uses Reversible Jump Markov Chain Monte Carlo (RJMCMC)^15,16^. The Bayesian formalism allows the inclusion of strong prior information such as the photophysics of the probe and the emitter density. RJMCMC allows classification uncertainty, i.e., uncertainty in the true number of emitters to be incorporated in the emitter location probability distribution. BAMF also couples background estimation and its uncertainty with inference of emitter locations and intensities. The result is a posterior probability distribution for emitter positions that considers both prior knowledge and sources of uncertainty that are often ignored.

Markov Chain Monte Carlo (MCMC) is a computationally efficient method for sampling from a multi-dimensional posterior probability distribution^17,18^. RJMCMC takes the concept of MCMC further and allows jumps between parameter spaces with different numbers or types of parameters. The acceptance probability for inter-space jumps is given by an extension of the Metropolis-Hasting formula (Supplementary Note 1)^15,16^, resulting in a chain that spends time in each space proportional to the posterior probability of that space. The histogram of the returned chain can be interpreted as a probability distribution for the parameters of interest, which in the multiple-emitter fitting problem are the emitters’ positions, whereas the other parameters and states can be marginalized out.

**Figure 1:**
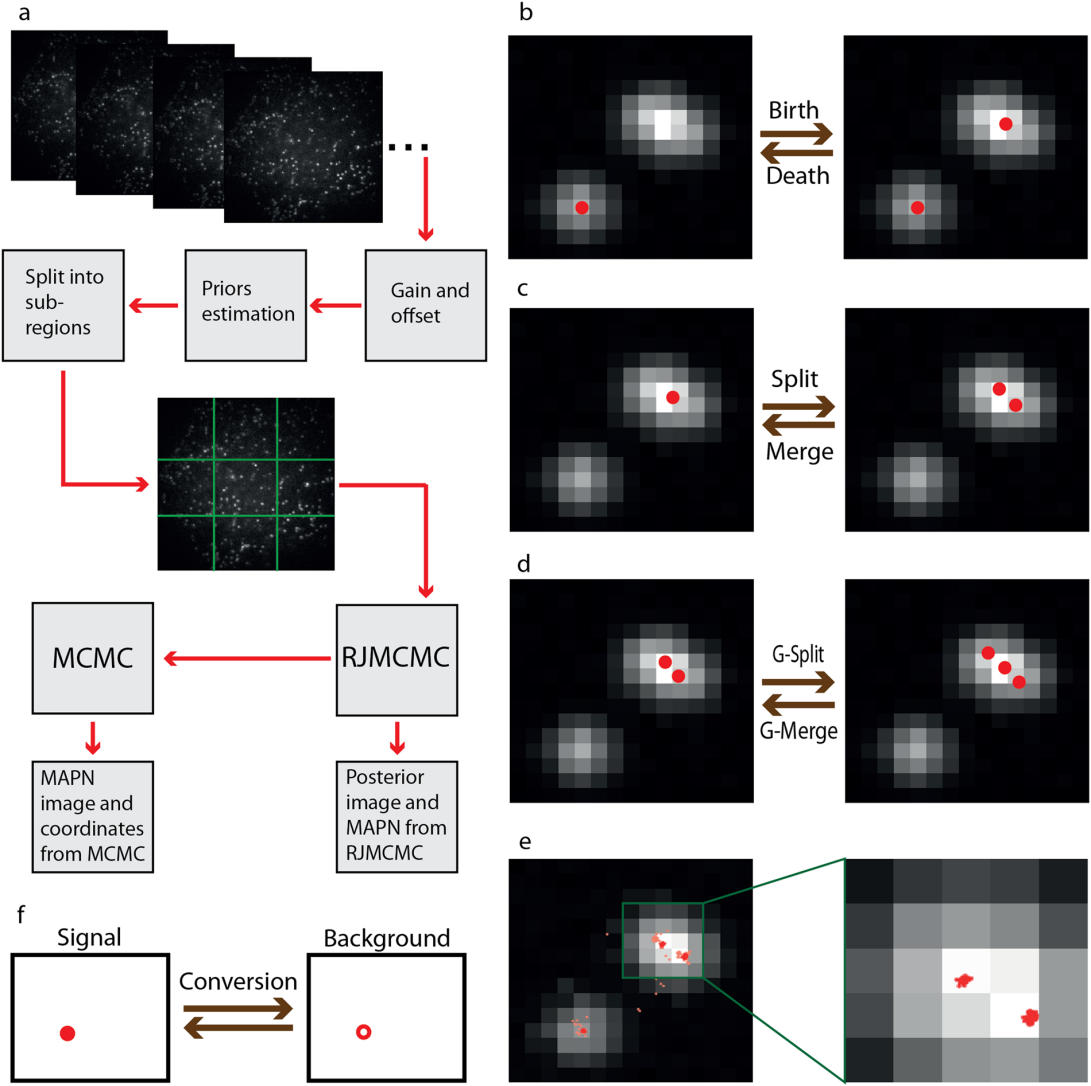
Data flow, jump types and the chain. (**a**) The data flow. Boxes show stages of the analysis. (**b**) From left to right, a new spot is detected through a birth event. From right to left, a death event is proposed and an existing emitter is removed. (**c**) From left to right, an existing emitter splits into two emitters. From right to left, two adjacent emitters merge into a single one. (**d**) From left to right, photons are taken from *N* existing emitters to make a new one. Right to left, an existing emitter breaks into N pieces which are added to *N* nearby emitters. G-split and G-merge stand for generalized split and generalized merge. (**e**) Left, the plot of a chain of 8,000 jumps, where lighter red shows the burn-in part and the darker red shows the chain after convergence. Right figure depicts the chain after convergence inside the green box. (**f**) The conversion jump uses the priors on intensities to classify the emitters as either a signal emitter, or an emitter used to model structured background.

The entire BAMF algorithm consists of several steps (Fig. 1a): (1) converting raw data to photon counts, (2) estimation of the intensity prior, (3) division of each image into subregions, (4) the core RJMCMC algorithm, (5) using the RJMCMC chain to initialize MCMC within the most probable space, (6) using the MCMC chain to calculate the parameters and their associated uncertainties, and (7) making the final reconstructions by removing the localizations in the overlapping areas of the subregions (Supplementary Video 1), and combining the results.

The RJMCMC step is used within a fitting subregion and calculates a posterior distribution to make inferences about a set of parameters. This requires both a likelihood model and prior distributions. The likelihood is calculated assuming a model consisting of a set of emitter positions, a PSF model (2D Gaussian^6,19^ or provided by the user), a tilted plane as unstructured background and Poisson statistics (Methods; PSF model and likelihood). The emitters model both apparent single emitters (signal) or structured background by using a collection of PSF-sized kernels (background) (Supplementary Fig. 5). Each parameter has a corresponding prior distribution that is given in Table 1.

**Table 1:** Table of BAMF parameters, their associated units and prior functions.

parameter	description	unit	prior
*x*, *y*	*X* & *Y* positions of emitter	pixel	uniform
*I*	number of photons from signal/background emitter	photon count	kernel density estimator/exponential
*N*	number of emitters	count	Poisson
*b*	offset of background plane	photon count	gamma
*a*_*x*_, *a*_*y*_	slopes of background plane along *X* & *Y* axes	photon count/pixel	normal
*l*	signal/background label for emitter	—	—

We allow three within-space moves (no change in number or type of emitters): 1) A single-emitter move that changes the position and intensity of one or more emitter; 2) A group move that makes correlated changes in two or more emitters, and 3) A background move, which changes the parameters of a tilted plane background model (Supplementary Note 1). We permit four pairs of reversible jump types between parameter spaces: (birth, death), (split, merge), (generalized split, generalized merge) and (signal, background) (Fig. 1b–d,f, Supplementary Figs 1, 5 and Supplementary Video 2–4). Birth (death) allows the addition (deletion) of an emitter anywhere in the model. Split and merge allows a split and merge between two emitters. Generalized split and merge splits or combines *N* emitters. This pair of jumps provides better mixing of the chain in dense regions of data, see Supplementary Fig. 8. Signal (background) converts an emitter from a PSF shaped kernel of a background structure to a detected emitter.

The output of the RJMCMC step is a parameter chain whose histogram can be interpreted as a probability density landscape of the emitters that considers all possible numbers and positions of emitters. For example, a single emitter appears as a blob-shaped feature in the histogram image of the chain of positions (Fig. 1e and Supplementary Video 3), where the width of the blob can be used to calculate the standard error for the position estimation. Combining the chains from all the subregions, we build the posterior image for each time frame and then add up the posteriors over all time frames to obtain the average posterior reconstruction image, which we simply call the posterior image hereafter. To generate a set of positions and uncertainties from the elements of the RJMCMC chain from the most probable model, the Maximum a Posteriori model of Number of emitters (MAPN), is either used directly or used to initialize a MCMC chain for the MAPN model. The results are used to calculate the positions and associated uncertainties. These returned localizations are then used to reconstruct an image. The posterior probability image includes uncertainty over the number of emitters, whereas the MAPN result can provide locations and standard errors that can be used in subsequent analysis (Supplementary Fig. 2).

## Results

To assess the performance of BAMF, we analyzed several types of synthetic data and compared the results with that from FALCON^10^, SRRF^11^ and single-emitter fitting^19^. Jaccard Index (JAC) and localization accuracy are two standard measures to assess the performance of SMLM fitting algorithms^20^. JAC is defined as the ratio of the number of the matched emitters from the sets of found and true emitters to the number of the emitters in the union of those two sets: JAC=$\frac{{\rm{ME}}}{{\rm{FE}}+{\rm{TE}}}$ where ME, FE and TE refer to the number of matched emitters, found emitters and true emitters, respectively (Methods; Tests on synthetic data).

**Figure 2:**
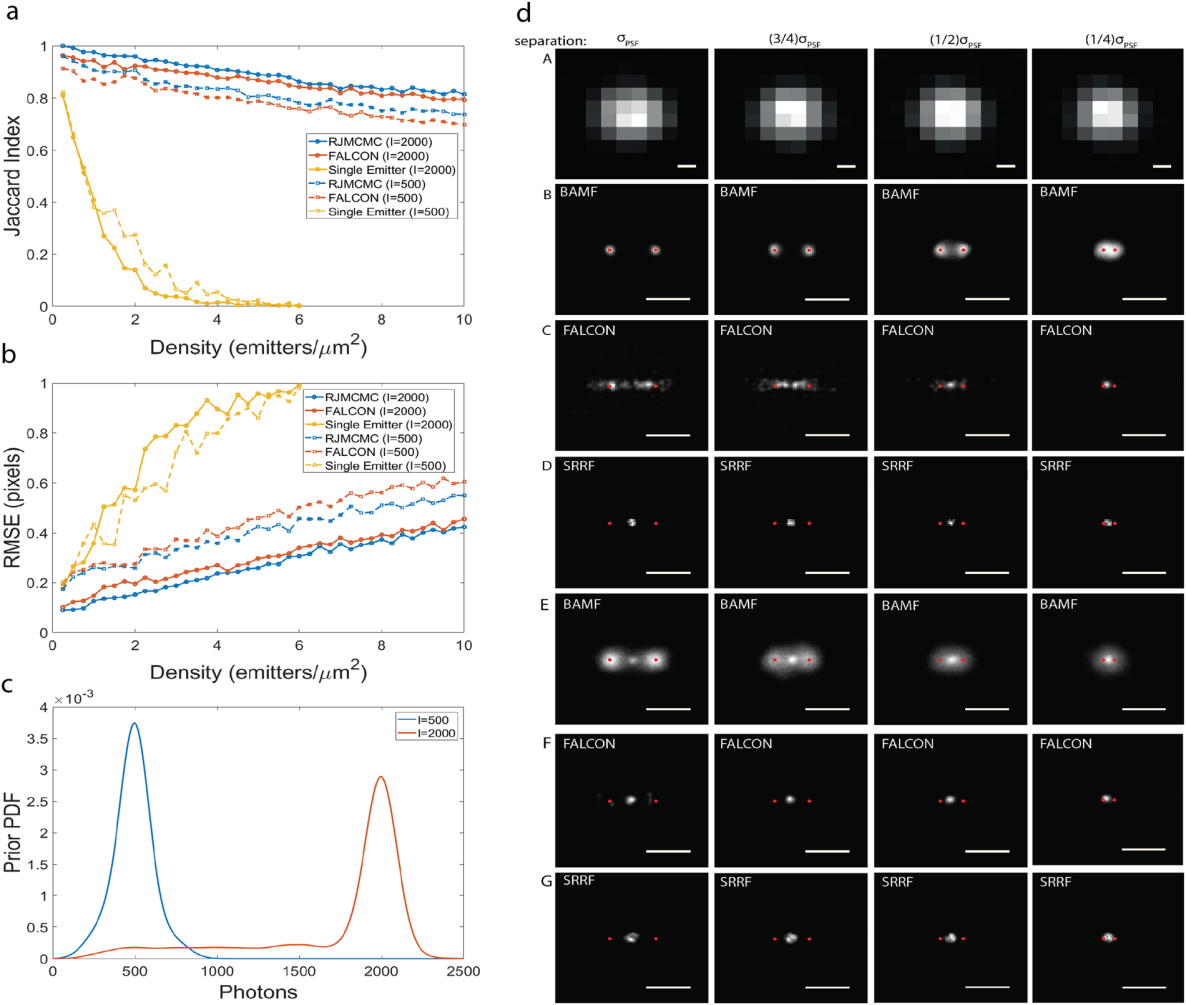
Jaccard index and localization error (accuracy). (**a**) Jaccard index, (**b**) localization errors for BAMF, FALCON and single-emitter fitting using 2,000 and 500 photons per frame. (**c**) The intensity priors used to analyze the data. To make these plots for BAMF, the MAPN was used from RJMCMC. (**d**) represents the ability of BAMF, FALCON and SRRF to distinguish two nearby emitters with different separations. Row A shows a frame of simulated data with different separations. Rows B, C and D result from emitters with 2,000 photons, and rows E, F and G show the results from 500 photon emitters. The BAMF super-resolved images are the posterior images containing all the possible models. The scale bars are σ_PSF_.

Localization accuracy is given by the mean distance between the matched pairs. We used the MAPN result to calculate JAC and accuracy for BAMF. JAC and accuracy were also calculated for FALCON and the single-emitter algorithm. SRRF returns images but not coordinates and therefore was not included. Figure 2 depicts JAC and localization accuracy for the three algorithms. BAMF outperforms the other approaches in both JAC and accuracy.

We compared the results of these algorithms on simulated sequences of data representing two nearby emitters with various separation distances and photons/frame. At 2,000 photons/frame, BAMF could distinguish two emitters down to a separation of about 0.25σ_PSF_, much better than FALCON and SRRF, which could only recognize the data as two close emitters when separated by more than σ_PSF_ (Fig. 2 and Supplementary Fig. 3). Here the prior information on emitter intensity helps constrains BAMF to the correct number of emitters. The trend continues to lower photon counts, however the effectively wider intensity prior distribution gives less constraint and the result is a mix of one and two emitter models.

We simulated and analyzed sequences of data with circular test structures of four different radii. FALCON returned more false emitters in the middle of circles where no true emitters reside. SRRF returned disks rather than rings. The single-emitter code returned a circle structure, but much fewer emitters (Supplementary Fig. 4).

To evaluate the ability of these methods to deal with structured background, we simulated a dataset with a static ring-like background structure along with in-focus emitters randomly distributed over a cross-like structure. BAMF and SRRF were able to distinguish signal and background structures, however, FALCON attempted to model the background with emitter locations (Supplementary Fig. 5). Microtubules in the data set from the SMLM Challenge were simulated in 3D and in some places they were out of focus for the 2D PSF. In those areas of the data, FALCON tried to model the out of focus emitters as a disperse collection of emitters. However, BAMF correctly modeled that area as structured background (Supplementary Fig. 7).

**Figure 3:**
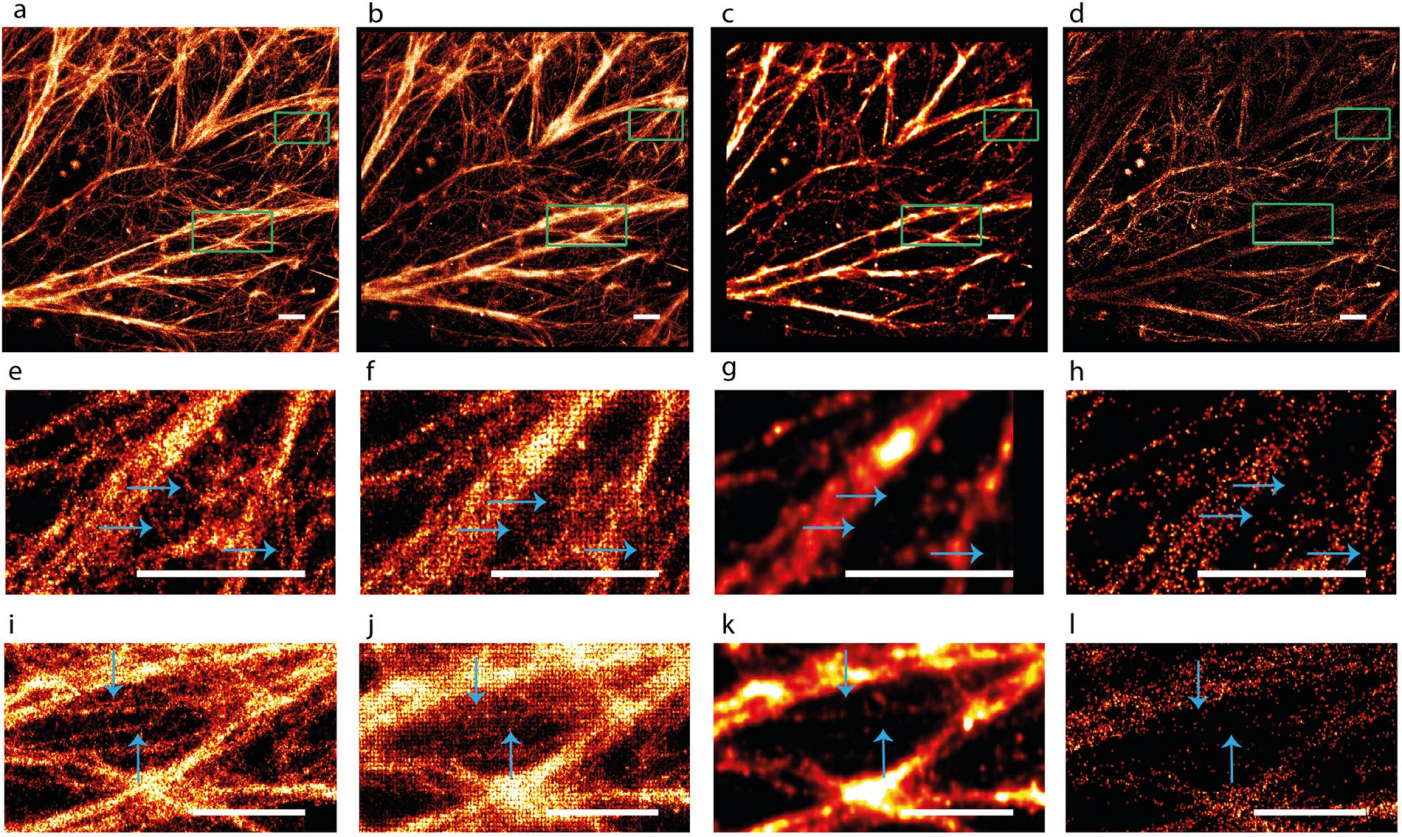
Reconstructions from BAMF, FALCON, SRRF and single-emitter fitting for dSTORM data of actin filaments. Reconstructions from (**a**) BAMF, (**b**) FALCON, (**c**) SRRF, (**d**) single-emitter fitting. The reconstructions for BAMF were made using the MAPN from MCMC. (**e**–**h**) Zoom of the top green square. (**i**–**l**) Zoom of the bottom green square. The blue arrows point to some fine details in the magnified regions. The scale bars are 1 μm.

**Figure 4:**
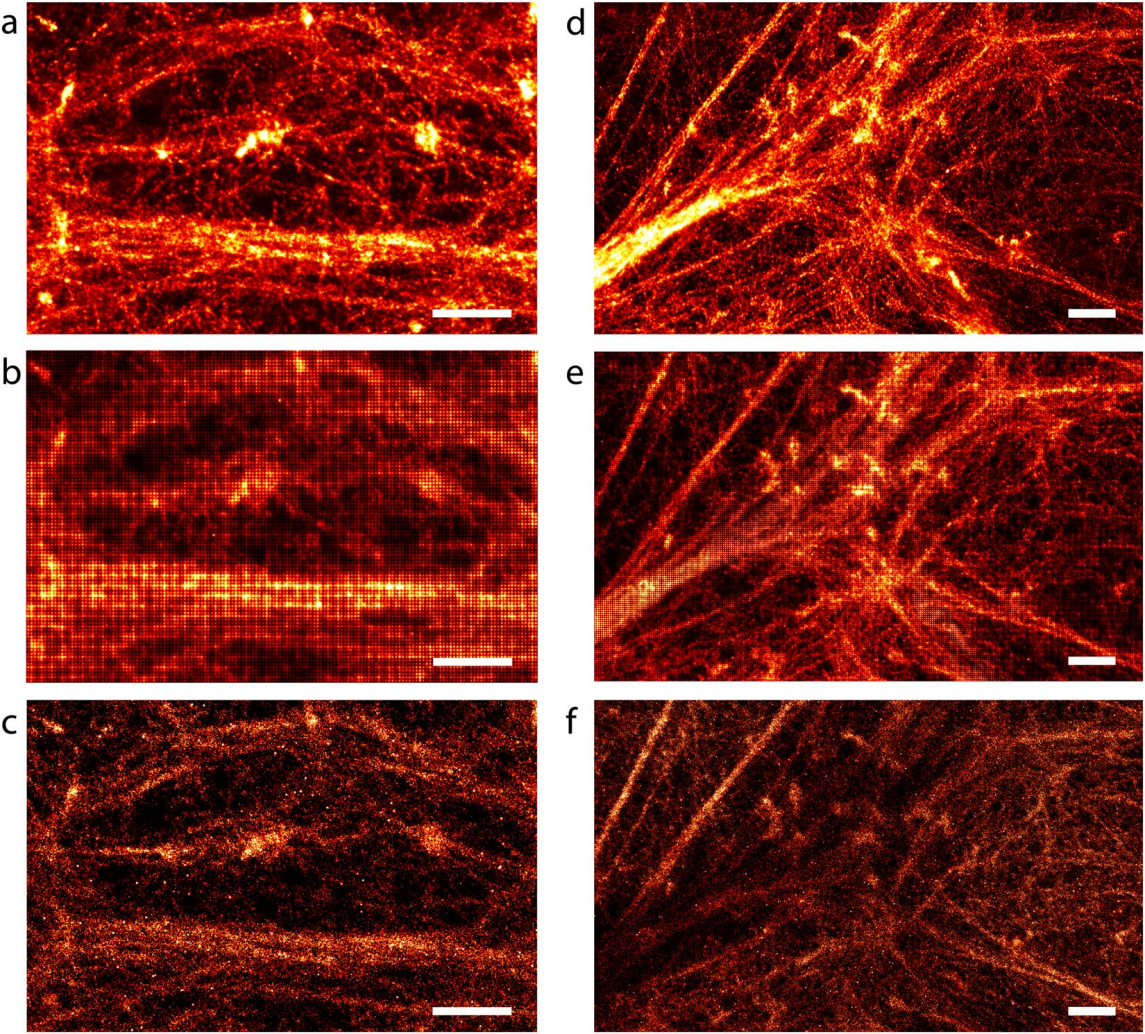
Selected magnified regions from BAMF (**a**,**d**), FALCON (**b**,**e**) and the single-emitter code (**c**,**f**) reconstructions in Supplementary Fig. 6. Each column represents the same regions from different fitting procedures. FALCON and single-emitter code do not reveal as many details as BAMF. FALCON has a grid-like artifact. **(f**) Shows the contrast inversion artifact for the single-emitter code. The scale bars are 1 μm.

We tested BAMF performance on dSTORM and DNA-PAINT experimental data (Figs 3 and 4, Supplementary Fig. 6), as well as simulated dense, low signal to noise microtubules data from the SMLM Challenge website^20^. Figure 3 shows the reconstructions from BAMF, FALCON, SRRF and the single-emitter code on actin imaged using dSTORM. In the two bottom rows in Fig. 3, the arrows show very fine actin filaments. BAMF reveals these actin filaments much better than the other algorithms. The single-emitter algorithm found much fewer localizations in those areas. FALCON does not show as many details as BAMF and has a grid-like artifact that is likely due to the grid used in the deconvolution step in FALCON. The reconstruction from SRRF is missing much of the fine detail. Figure 4 and Supplementary Fig. 6 show similar trends in the results from BAMF, FALCON and single-emitter algorithm on actin imaged using DNA-PAINT. Supplementary Fig. 7 displays the resulting reconstructions from BAMF and FALCON for the simulated microtubules data from SMLM Challenge website. The green arrows point to two very close microtubules, which visually are more distinguishable in the reconstruction from the BAMF algorithm.

## Discussion

The BAMF algorithm takes advantage of prior information to improve the classification of the number of emitters and includes the effect of uncertainty in both classification of number of emitters and the background structure. BAMF generates both a posterior image that contains all sources of uncertainty and a MAPN result that provides coordinates and standard errors of the most probable model. BAMF outperforms other common fitting models both quantitatively on synthetic data and subjectively on experimental data. The BAMF algorithm is also able to detect and localize emitters very close to the edges of the frame as opposed to other approaches, Fig. 3(a–c). Deep learning methods have recently been employed to address the fitting problem in super-resolution microscopy^13,14^. Although these methods are computationally fast during analysis, they require training, which has to be done independently for different microscopes and/or experimental conditions. Deep learning approaches have so far returned only images rather than localizations, and do not provide a measure of the uncertainty in the localizations which can be required for further analysis.

BAMF produced superior results in comparison to the other methods tested in both synthetic and experimental data. BAMF has a particular advantage for closely spaced emitters where the intensity prior helps constrain the model as can be seen in Fig. 2d and Supplementary Fig. 3. When emitters are spaced ~ σ_PSF_ or further on average, such as emitters spaced randomly with a uniform distribution as used for the JAC calculations, there is a smaller advantage over FALCON, which favors sparse models.

BAMF couples a structured background estimation together with emitter localization, which allows the algorithm to detect, model, and essentially remove any heterogeneous background, such as might arise from parts of the sample that are out of focus. The uncertainty of the background estimation is inherently propagated to the uncertainty in emitter locations. However, we note that in practice localization precisions may not be largely affected (Supplementary Fig. 11). SRRF uses temporal information to selectively analyze blinking emitters and does a good job at background rejection (Supplementary Fig. 5g). FALCON uses a background estimation step in pre-processing, but this fails to remove highly structured background and this background is modeled as true emitters in the final result (Supplementarys Figs 5h, 7c).

BAMF is somewhat computationally intensive, where the computational cost rises almost linearly with increasing density of emitters (Supplementary Fig. 12). It took ~8 hours to analyze 12,000 frames of 128 × 128 pixels in order to construct Fig. 3 using a PC with an i7, 3.64 GHz CPU. The algorithm can be significantly sped up by implementing critical portions on a GPU, where subregions would be processed independently in parallel.

The algorithmic framework of BAMF could be extended in a straightforward manner to 3D imaging by using engineered PSFs and a likelihood model that includes an axial position parameter. BAMF analyzes data frame-by-frame in an independent manner. BAMF could also be extended to include temporal information by analyzing *x*, *y*, *t* data cubes using additional parameters for the start and end of blinking events.

## Methods

### PSF model and likelihood

Photons from a single emitter have an approximate spatial Gaussian distribution on the camera, where (1) gives photon counts based on this approximation. For cases where the Gaussian function is not a reasonable approximation, the PSF can be acquired experimentally and employed to calculate the likelihood numerically (Supplementary Note 4). The integral of the Gaussian distribution over the *k*th pixel gives the average number of photons from the *i*th emitter in that pixel.

1 $${\Delta }_{k,i}=\frac{{I}_{i}}{2\pi {{\sigma }_{{\rm{PSF}}}}^{2}}{\int }_{{x}_{k}-0.5}^{{x}_{k}+0.5}{\int }_{{y}_{k}-0.5}^{{y}_{k}+0.5}\exp [\frac{{(x-{x}_{i})}^{2}+{(y-{y}_{i})}^{2}}{2\pi {{\sigma }_{{\rm{PSF}}}}^{2}}]dydx$$

where *Δ*_k,i_, *σ*_PSF_, *I*_i_, *x*_k_, *y*_k_, *x*_i_ and *y*_i_ are, respectively, the number of the photons in the *k*th pixel from the *i*th emitter, the half width of the Gaussian distribution, the total number of the photons from the emitter, the center of the *k*th pixel, and the position of the emitter.

The total photon count in the *k* th pixel is the sum of the photons from all the existing emitters and the background.

2 $${\lambda }_{k}(N)={a}_{x}x+{a}_{y}y+b+\mathop{\sum }\limits_{i=1}^{N}{{\Delta }}_{k,i}$$

where *λ*_k_ and *N*, respectively, denote the total number of the photons in the *k*th pixel, and the number of emitters. The background noise is modeled by a slightly tilted plane with offset *b* and slopes *a*_*x*_, *a*_*y*_ along the *X* and *Y* axes, respectively. Equation (2) yields the expected photon counts for the pixel *k* for a fixed exposure time. Consequently, the number of the photons in pixel *k* has a Poisson distribution

3 $${P}_{k}(D|\theta )=\frac{{\lambda }_{k}{(N)}^{{D}_{k}}{e}^{-{\lambda }_{k}(N)}}{{D}_{k}!}$$

where *θ* represents the set of the parameters $(\theta =(\overrightarrow{x},\overrightarrow{y},\overrightarrow{I},N,b,{a}_{x},{a}_{y},l))$, Table 1. Note that the signal and background emitters contribute to the likelihood in the same manner so the likelihood is not affected by the labeling or classification parameter, *l*. *D* stands for the data, which is a two dimensional array of pixels whose values are the number of photons captured by the camera. These photons can come from blinking emitters or (structured) background. *D*_*k*_ selects the *k* th pixel in *D*. Due to the independence of the pixels, the likelihood of the frame is given by the product of the likelihoods of all the pixels in that frame [6, 17].

4 $$P(D|\theta )=\prod _{k}{P}_{k}(D|\theta )$$

### Priors and posterior

RJMCMC can be used to implement a Bayesian approach that samples from the posterior of a system in order to learn about that system. The posterior is proportional to the product of the likelihood and priors:

5 $$P(\theta |D)=\frac{P(D|\theta )P(\theta )}{P(D)}$$

where *P*(*D*) is called the evidence. Evidence is the normalization coefficient of the posterior.

6 $$P(D)=\int P(D|\theta )P(\theta )d\theta $$

We employ RJMCMC to estimate the position and intensities of the emitters, the number of the emitters, the offset background and its slopes, and therefore their priors have to be included in the calculations. We take the prior on the positions, (*x*, *y*), to be a uniform distribution over the subregions. Because there might be some emitters outside but still close enough to its edges so that portions of the PSFs are still observable on the subregion, we allow the detection of emitters that are located up to 2 pixels away from the edges outside the subregion (this can be modified by user). Hence the prior is a uniform distribution over this extended range. The number of the emitters, *N*, inside the region of interest has a Poisson distribution with the mean value *ρW*^2^, where *ρ* is the density of emitters per pixel given by the user as an input, and *W* is the width of the region of interest in pixels. The slopes of the offset background, (*a*_*x*_, *a*_*y*_), have a normal distribution as the prior where the center and width are, respectively, fixed at zero and one because the tilt of the offset background plane over the range of a small subregion are not typically larger than 1 photon per pixel.

We implement empirical priors for several of the parameters using a fast single-emitter fitting code^19^ to find the priors or prior parameters. A MATLAB library consisting of several methods is provided along with the BAMF code that calculates these priors; see Supplementary Note 3 and Table 1. We used a gamma distribution as the prior for offset background, *b*, where the parameters were estimated from the values returned by the single-emitter code. The intensity, *I*, distribution of emitters heavily depends on several conditions such as the on and off rate of the emitters, the labeling method, etc. Therefore, it is not feasible to consider a specific functional form as prior intensity distribution for general data. Because of that, the signal intensity prior is given as a smooth curve, obtained via a smoothed kernel density estimator fit to the intensity values returned by the fast single-emitter fitting algorithm. We utilize an exponential distribution for the background intensity prior. The mean of the exponential prior is the mean of the intensity of the signal priors divided by a scaling constant provided by the user.

### BAMF’s parameters

For the moves in position, intensity, and offset background, jumps were selected from zero-mean normal distributions with the sigma ranging from 0.05 to 0.1 pixel, 5 to 10 photons, and 1 photon, respectively. For the burn-in chain, 3,000 jumps and jump probabilities of (Р_In-model_, Р_Birth_, Р_Death_, Р_Split_, Р_Merge_, Р_G-split_, P_G-merge_, Р_Conversion_) = (0.3, 0.1, 0.1, 0.1, 0.1, 0.1, 0.1, 0.1) were used, while for the post-burn-in chain, we had 2,000 jumps and jump probabilities of (Р_In-model_, Р_Birth_, Р_Death_, Р_Split_, Р_Merge_, Р_G-split_, P_G-merge_, Р_Conversion_) = (0.4, 0.05, 0.05, 0, 0, 0.15, 0.15, 0.2). We allowed more between-model jumps in the burn-in portion because the chain needs to explore different models and detect new emitters. More within-model jumps are proposed after the burn-in portion to fine tune the parameters for the detected emitters in the burn-in portion. For the JAC measurements and two-emitter simulations, the high density of emitters required 20,000 jumps and 10,000 jumps for burn-in and post-burn-in respectively, to guarantee the chain convergence, see Supplementary Fig. 9. The subregion size used was 16 × 16 pixels.

### Chain mixing and convergence

The jump sizes for each parameter in the RJMCMC step were adjusted to yield an acceptance rate of 25% to 50% for within-model jumps (Supplementary Note 1). To further evaluate the mixing and convergence of the RJMCMC chain with the selected parameters, we ran the BAMF algorithm two times for the same 20 random subregions and then generated posterior images of the accepted jumps inside the two chains. The image cross correlations of the timewise corresponding jumps from the two chains were calculated and averaged over the 20 subregions. When the calculated cross correlations approach one over the used number of jumps, it demonstrates that the chain is converging and mixing well (Supplementary Fig. 9).

To evaluate parameter convergence, we simulated a 16 × 16 sub-region of super-resolution data containing 5 signal emitters with average intensity and PSF size of 2000 photons and 1.2 pixels. The data was processed in the presence and absence of structured background, Supplementary Fig. 10.

### Implementation

Image pre-processing and computational analyses were performed in MATLAB by employing the image processing, statistics and machine learning and parallel toolboxes (MathWorks Inc.). The C++ -codes for RJMCMC were compiled into mex-files that could be called from inside MATLAB. All codes were CPU based and were parallelized using the MATLAB parallel computing toolbox. The single-emitter code was implemented on GPUs using CUDA codes compiled into ptx-files that could be called inside MATLAB. An i7, 3.64 GHz CPU with a GTX 750 GPU was used to process the simulated data and part of the experimentally acquired data. Part of the experimental data was also analyzed in a cluster employing 16 core Intel Xenon 2.6 GHz CPUs, available at the UNM Center for Advanced Research Computing (CARC).

### Synthetic data generation

To generate synthetic data, emitters were placed in random positions with the uniform density ρ, except where mentioned. A trace of the blinking events of each emitter was produced using the duty cycle parameters, *k*_on_ and *k*_off_, which are respectively the rate of emitters going from off to on and on to off, such that the density of the on-emitters is proportional to the ratio of *k*_on_ to *k*_on_ + *k*_off_. To imitate realistic conditions, random times for the emitters to turn on and on-durations were chosen, using exponential distributions with mean values of *k*_on_ + *k*_off_ and *k*_on_, respectively. Next, a uniform background was added to the generated data and corrupted with Poisson noise.

### Tests on synthetic data

Jaccard index (JAC) and accuracy were calculated by making use of synthetic data generated in a region of 24 × 24 pixels, where 2 pixels at the edges were left empty, with the pixels of width 100 nm. A ground truth of 1,000 emitters per μm^2^ was generated and the duty cycle parameters were adjusted to provide a desired final per-frame density. 40 sequences of 100 frames of data were generated with an average density of on-emitters ranging from 0.25 to 10 emitters per μm^2^ over a uniform background. The width of the PSF and the offset background were, respectively, 1.2 pixel and 20 photons. The intensity of the emitters that were on during an entire frame exposure was 2,000 photons per frame and less if they were on for a fraction of the exposure time. We used *k*_*off*_ = 0.4/frame while *k*_*on*_ was calculated from ${{\rm{\rho }}}_{{\rm{on}}} \sim {\rm{\rho }}\frac{{{\rm{k}}}_{{\rm{on}}}}{{{\rm{k}}}_{{\rm{on}}}+{{\rm{k}}}_{{\rm{off}}}}$, where *ρ* and *ρ*_*on*_ are, respectively, the density of ground truth emitters and the density of on-emitters.

The localization accuracy was measured by the root mean square error (RMSE) to the true locations. In order to calculate JAC, matched pairs between the MAPN result (used directly from RJMCMC chain) and the true emitters were found. To discover the pairs of the matched emitters, the cost matrix of the found emitters with the true emitters were minimized using the Hungarian algorithm^21^, and those pairs where the corresponding cost element was smaller than the PSF size (1.2 pixel) were used in the JAC^20^.

For the synthetic circles, data sequences of 2,000 frames with a size of 10 × 10 pixels were produced with circles of radii of 0.416σ_PSF_, 0.625σ_PSF_, 0.833σ_PSF_ and 1.041σ_PSF_, and with a PSF width, mean intensity and background of 1.2 pixel, 2,000 photons and 20 photons, respectively. Uniformly distributed emitters at 1,000 per μm were used to generate the circles and then by adjusting the duty cycle parameters brought to an average density of 4.5 on-emitters per frame. The localizations returned by the single-emitter code, FALCON and BAMF were used to reconstruct the final images of circles. SRRF does not return any localizations but does return a reconstruction which was included in Supplementary Fig. 4. Since FALCON does not return any localization accuracy, the same accuracy (σ = 0.06 pixel, which is the mode of localization accuracy returned by BAMF) was used to reconstruct the final images for the three algorithms. The reconstructions from BAMF with localization accuracy better than 0.25 pixel are also included in Supplementary Fig. 4.

For the two emitters test, two sets of 100 frames of data were synthesized using two constantly on emitters for each separation. The PSF width, intensity and background were 1.2 pixel, 500 or 2,000 photons (representing dim and bright emitters in empirical data), and 20 photons respectively. The priors used for JAC and accuracy measurements with average intensities of 500 (dim) and 2,000 (bright) photons (Fig. 2) were employed because the two emitters were constantly on and heavily overlapped and the single-emitter code was not able to estimate their intensities. This is the only exception to the protocol described in the supplement to obtain the intensity priors.

A sequence of data of 2,000 frames with size of 32 × 32 pixels was generated for the test of separation of signal emitters from structured background (Supplementary Fig. 5). The structured background was produced by placing 18 constantly on-emitters on positions equally spaced on a ring with a radius of 10 pixels. The PSF size and intensity of these emitters were 1.5 pixel and 400 photons per frame. For the signal, we synthesized 600 uniformly distributed emitters per μm^2^ inside a cross with PSF size and average intensity of 1.2 pixel and 2,000 photons and obtained 6.5 activated emitters per frame by tuning the duty cycle parameters. The final data set was produced by adding the two synthesized data sets with an offset background of 20 photons corrupted with Poisson noise. To compare the returned precisions by BAMF in the presence and absence of structured background, we processed the same simulated data set but the structured background was not included in this data set, Supplementary Fig. 11.

To evaluate the computational cost of the BAMF algorithm, 40 sequences of data of size 2.4 × 2.4 μm2 and 20 frames were generated where the density of the emitters started from 0.25 emitters/μm^2^ and incremented by 0.25 up to 10 emitters/μm^2^. They were processed by BAMF using 5,000 jumps per frame and the computational cost was calculated by averaging the time of each sequence over the number of frames in the sequences, the area of the frame and the number of jumps, Supplementary Fig. 12.

### Experimental data analysis

#### dSTORM actin data

The single-emitter code was used to find the PSF size and the prior distribution for the photons/emitter/frame (intensity) parameter. The PSF size was used for BAMF and FALCON.*DNA-PAINT actin data:* The single-emitter code was used to find the PSF size and the prior distribution. The provided library function *findPSF_SMA* (Supplementary Note 3) was used to calculate the PSF for BAMF. The found PSF size was used for FALCON.

The returned coordinates from the single-emitter code and BAMF were then filtered, eliminating the localizations with high uncertainties, in order to reconstruct the final images. FALCON does not return any uncertainty and hence the returned coordinates were used to produce the reconstructions directly. SRRF returns neither coordinates nor uncertainties, so only the returned reconstructions from it were used.

### SMLM Challenge data analysis

We used the MT4.N2.HD (2D) data from the SMLM challenge website^20^. This data set is simulated over 3020 frames with high emitter density and a low signal to noise ratio. To analyze this data, we used the BAMF library that uses the single-emitter code to find the intensity priors. For both FALCON and BAMF, we used information from the SMLM challenge website to adjust the required parameters.

### Cell lines and reagents

HeLa cells were cultured in Dulbecco’s Modified Eagle Medium (Life Technologies # 10313-v021) supplemented with 10% cosmic calfserum (HyClone), 1% penicillin-streptomycin (ThermoFisher, Cat # 25030081), and 2 mM L-glutamine at 37 °C and 5% CO_2_. Actin microfilaments were labeled with 0.56 μM Alexa Flour^TM^ 647 Phalloidin (ThermoFisher Scientific, A22287) diluted in PBS.

### Cell fixation and labeling

#### dSTORM actin imaging

All labeling and washing steps were carried out at room temperature unless stated otherwise. Cells were seeded onto #1.5 coverslip glass 6 well chambers (LabTek) to adhere for 24 h. Cells were fixed for 1 hour in a 3% Glyoxal + 20% Ethanol + 0.75% Ascetic Acid in DI water^22^. pH was adjusted to 5 by adding drops of 1 M NaOH. Cells were washed 2x in PBS and kept in NaBH_4_ for 10 min to reduce background fluorescence, followed by 2x wash with PBS. To quench reactive crosslinkers, the samples were kept in 10 mM Tris for 10 min, followed by 2 washes with PBS. Next, samples were blocked in 5% bovine serum albumin (BSA) + 0.05% Triton X-100 for 15 min. Finally, samples were washed 1x with PBS and labeled with 0.56 μM Alexa Flour^TM^ 647 Phalloidin for 4 hours. *DNA-PAINT actin imaging*: Cos7 cells were fixed and labeled with an actin-binding affimer linked to a DNA-PAINT docking strand as described previously^23^.

### Super-resolution imaging

#### dSTORM actin imaging

Imaging was performed in a standard dSTORM imaging buffer^24^ with an enzymatic oxygen scavenging system and primary thiol: 50 mM tris, 10 mM NaCl, 10% w/v glucose, 168.8 U/ml glucose oxidase (Sigma #G2133), 1404 U/ml catalase (Sigma #C9322), and 60 mM 2-aminoethanethiol (MEA), pH 8.5. To mount the samples prepared on 25 mm coverslips, an Attofluor cell chamber (Life Technologies, A-7816) was used and ~1.5 ml of dSTORM imaging buffer was added. To prevent oxygen permeation into the buffer, a clean 25 mm coverslip was used to seal the chamber. The sample was mounted on the stage of the microscope with a custom designed chamber holder. The imaging system was built on an inverted microscope (IX71, Olympus America Inc.). An *xyz* piezo stage (Mad City Labs, Nano-LPS100) mounted on a *x-y* manual stage was installed on the microscope for cell location and brightfield registration. A mounted LED with the wavelength of 850 nm (M850L3, Thorlabs) was used for brightfield illumination. Brightfield images was collected on a complementary metal-oxide semiconductor (CMOS) camera (Thorlabs DCC1545M) after reflecting by a short-pass dichroic beam splitter (FF750-SDi02, Semrock) and passing through a single-band bandpass filter (Semrock, FF01-835/70-25). A 638 nm laser was used (collimated from a laser diode, Thorlabs, L638P200) coupled into a single mode fiber and focused onto the back focal plane of the 1.49 NA objective lens (UAPON 100XOTIRF, Olympus America Inc.). Emission for super-resolution data was collected through a short-pass dichroic beam splitter (Semrock, FF750-SDi02) and a single-band bandpass filter (Semrock, FF624-Di01) on an iXon 860 electron-multiplying charge-coupled device (EM CCD) camera (Andor Technologies, South Windsor, CT). All the instruments were controlled by custom-written software in MATLAB (MathWorks Inc.). Imaging was performed with TIRF illumination. Images were acquired at 5 ms exposure time for a total of 12,000 frames. Brightfield registration was performed to correct for drift after every 3,000 frames as previously described^25^. *DNA-PAINT actin imaging*: Data was collected with 50 ms exposure time for 300k frames using 800 pM P1 imager strand concentration and 3.3 kW/cm^2^ laser power at 561 nm^23^.

## Data Availability

The datasets generated during and/or analyzed during the current study are available from the corresponding author on reasonable request.

## Supplementary information

Bayesian Multiple Emitter Fitting using Reversible Jump Markov Chain Monte Carlo

The algorithm package

Supplementary Video 1

Supplementary Video 2

Supplementary Video 3

Supplementary Video 4
