# Supplementary material for: Bayesian Multiple Emitter Fitting using Reversible Jump Markov Chain Monte Carlo
Source: Sci Rep. 2019 Sep 24;9:13791. doi: 10.1038/s41598-019-50232-x (PMC6760159; doi:10.1038/s41598-019-50232-x)
Supplement: Supplementary file 1 — Bayesian Multiple Emitter Fitting using Reversible Jump Markov Chain Monte Carlo [file 41598_2019_50232_MOESM1_ESM.pdf]

Supplementary Information
Bayesian Multiple Emitter Fitting using Reversible Jump Markov Chain Monte Carlo

Mohamadreza Fazel[1], Michael J. Wester[2], Hanieh Mazloom-Farsibaf[1], Marjolein B. M. Meddens[1], Alexandra S. Eklund[3,4], Thomas Schlichthaerle[3,4], Florian Schueder[3,4], Ralf Jungmann[3,4] and Keith A. Lidke[1]

[1] Department of Physics and Astronomy, University of New Mexico Albuquerque, New Mexico, USA.
[2]Department of Mathematics and Statistics, University of New Mexico, Albuquerque, New Mexico, USA.
[3]Department of Physics and Center for Nanoscience, Ludwig Maximilian University, Munich, Germany.
[4]Max Planck Institute of Biochemistry, Martinsried, Germany.

August 27, 2019

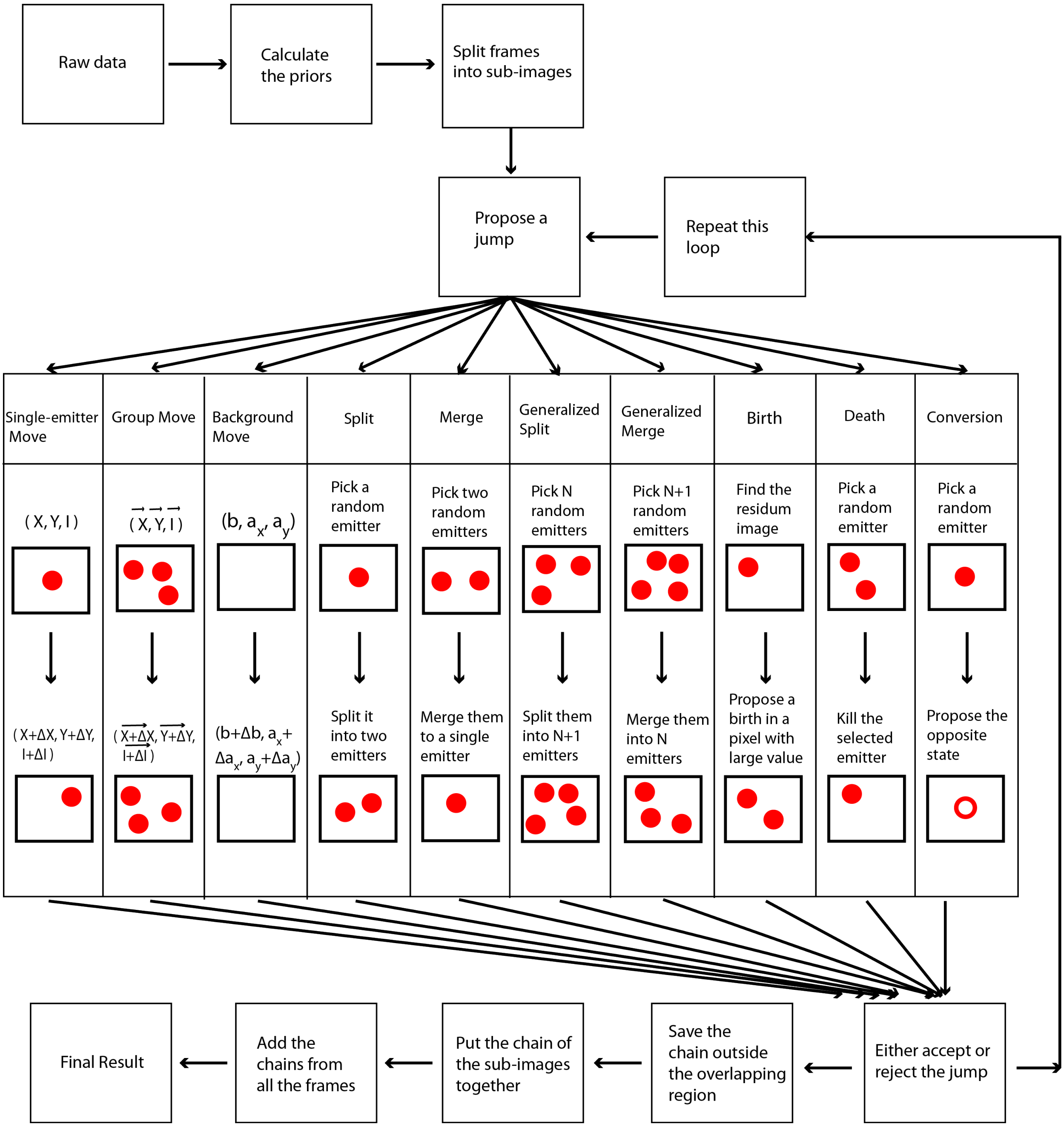

Supplementary Figure 1: BAMF concept: Schematic description of the BAMF algorithm. The flow of the data is described and the ten different jump types used in RJMCMC are depicted in the large box.

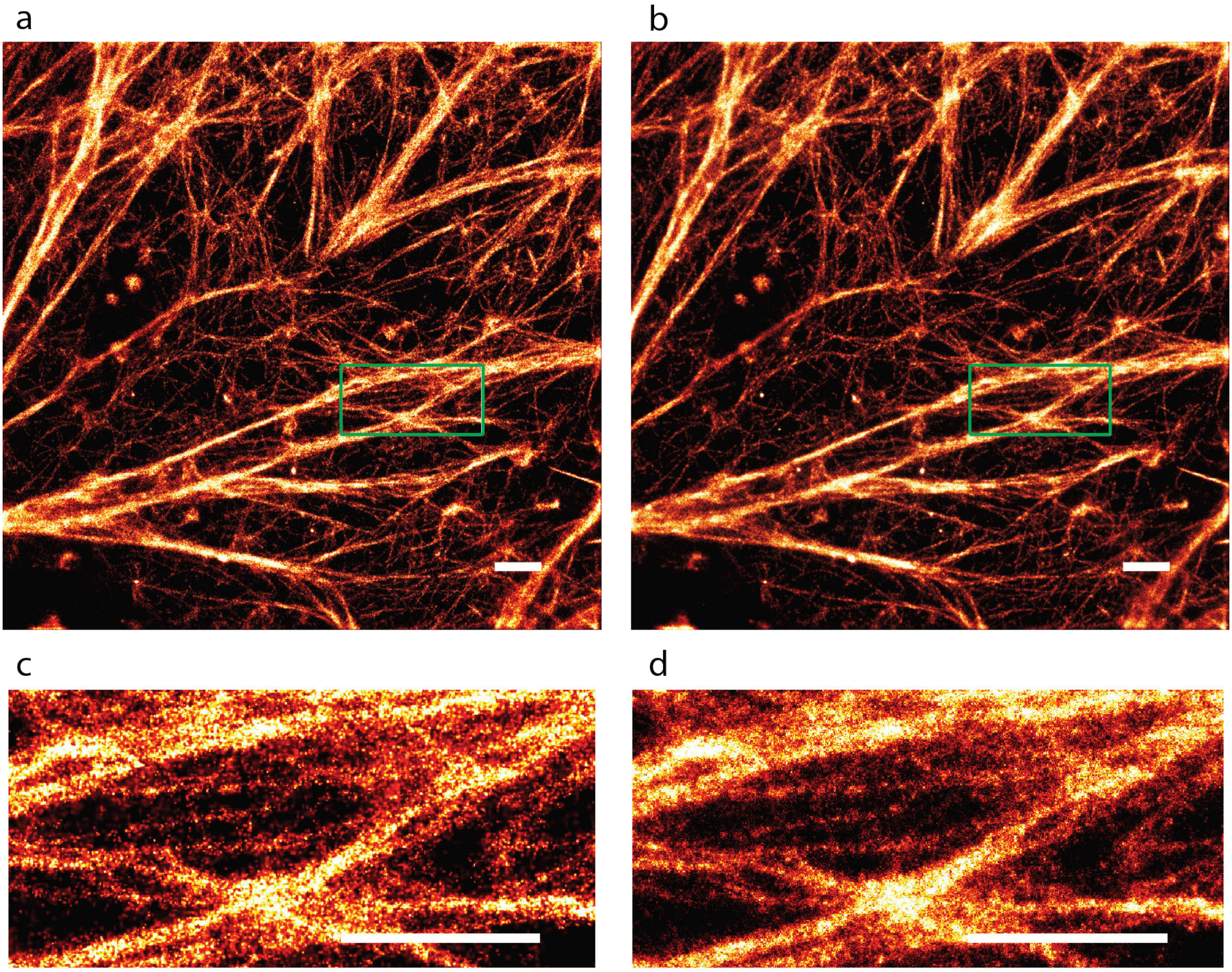

Supplementary Figure 2: Comparison of MAPN image and posterior image for dSTORM data of actin filaments from Hela cells. (a) and (b), respectively, show the MAPN and posterior images of the actin filaments from BAMF algorithm. (c) and (d) are zooms of the green squares. The scale bars are 1 μm.

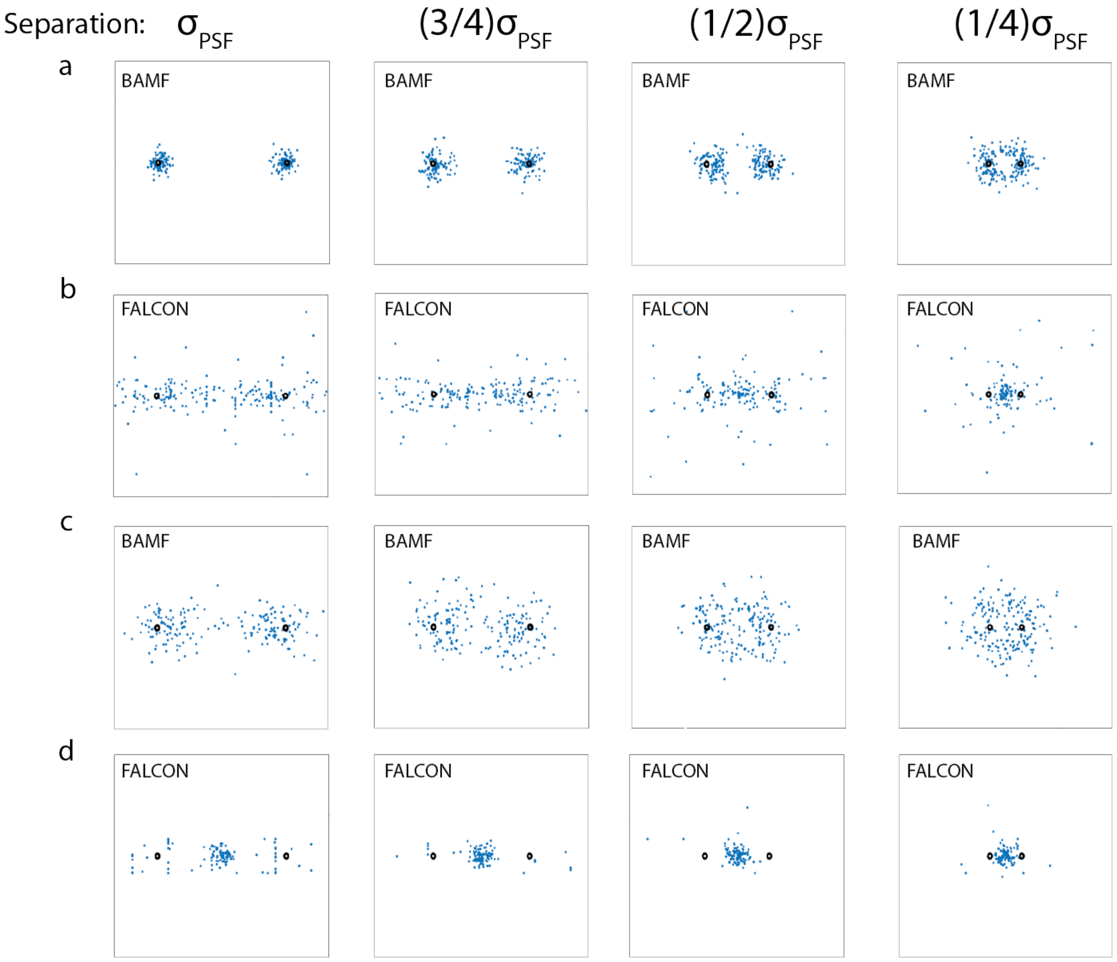

Supplementary Figure 3: Plot of found positions for two nearby emitters with different separations and intensities. The black circles represent the true locations while the blue dots stand for the found positions by BAMF and FALCON. The true locations of the first, second, third and fourth columns respectively, have separations of σ_{PSF}, $\frac{3}{4}\sigma_{\text{PSF}}$, $\frac{1}{2}\sigma_{\text{PSF}}$ and $\frac{1}{4}\sigma_{\text{PSF}}$, where $\sigma_{\text{PSF}} = 1.2$ pixels. Rows (a) and (c) show the plots of MAPN from RJCMC for intensities of 2000 and 500 photons, respectively. Rows (b) and (d), respectively, depict the results from FALCON for intensities of 2000 and 500 photons.

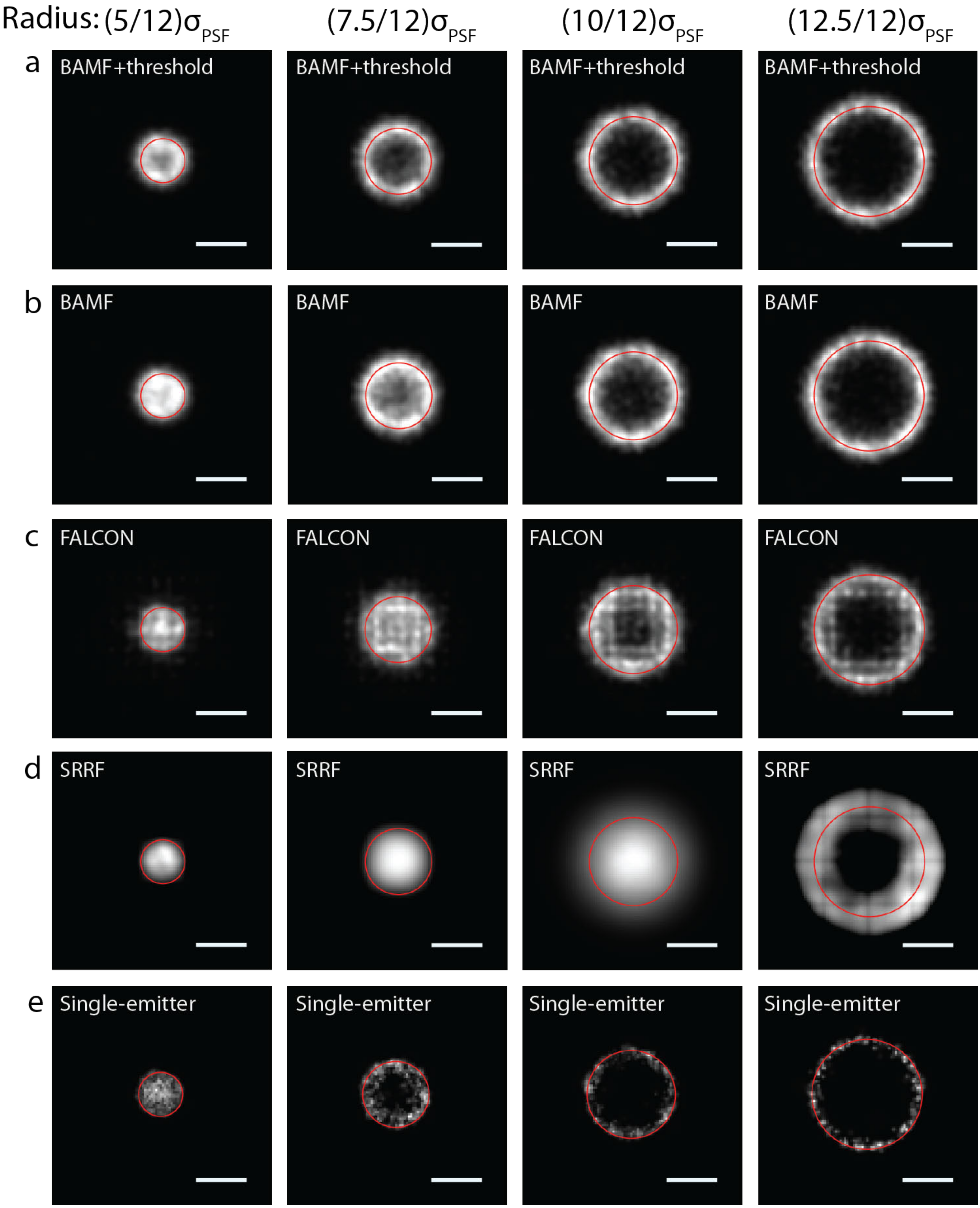

Supplementary Figure 4: Reconstructions for circles of emitters from FALCON, SRRF and the single-emitter code are depicted in rows (c), (d) and (e), respectively. BAMF reconstructions are shown in rows (a) and (b), which are MAPN from MCMC. Row (b) represents the circles reconstructed using all the returned localizations, while row (a) shows reconstructions from localizations with accuracy better than 0.25 pixel. The circles in the first, second, third and fourth columns, respectively, have radii of $\frac{5}{12}\sigma_{\mathrm{PSF}}$, $\frac{7.5}{12}\sigma_{\mathrm{PSF}}$, $\frac{10}{12}\sigma_{\mathrm{PSF}}$ and $\frac{12.5}{12}\sigma_{\mathrm{PSF}}$. The average density of on-emitters is 4.5 emitters per frame. The scale bars are σ_{PSF}.

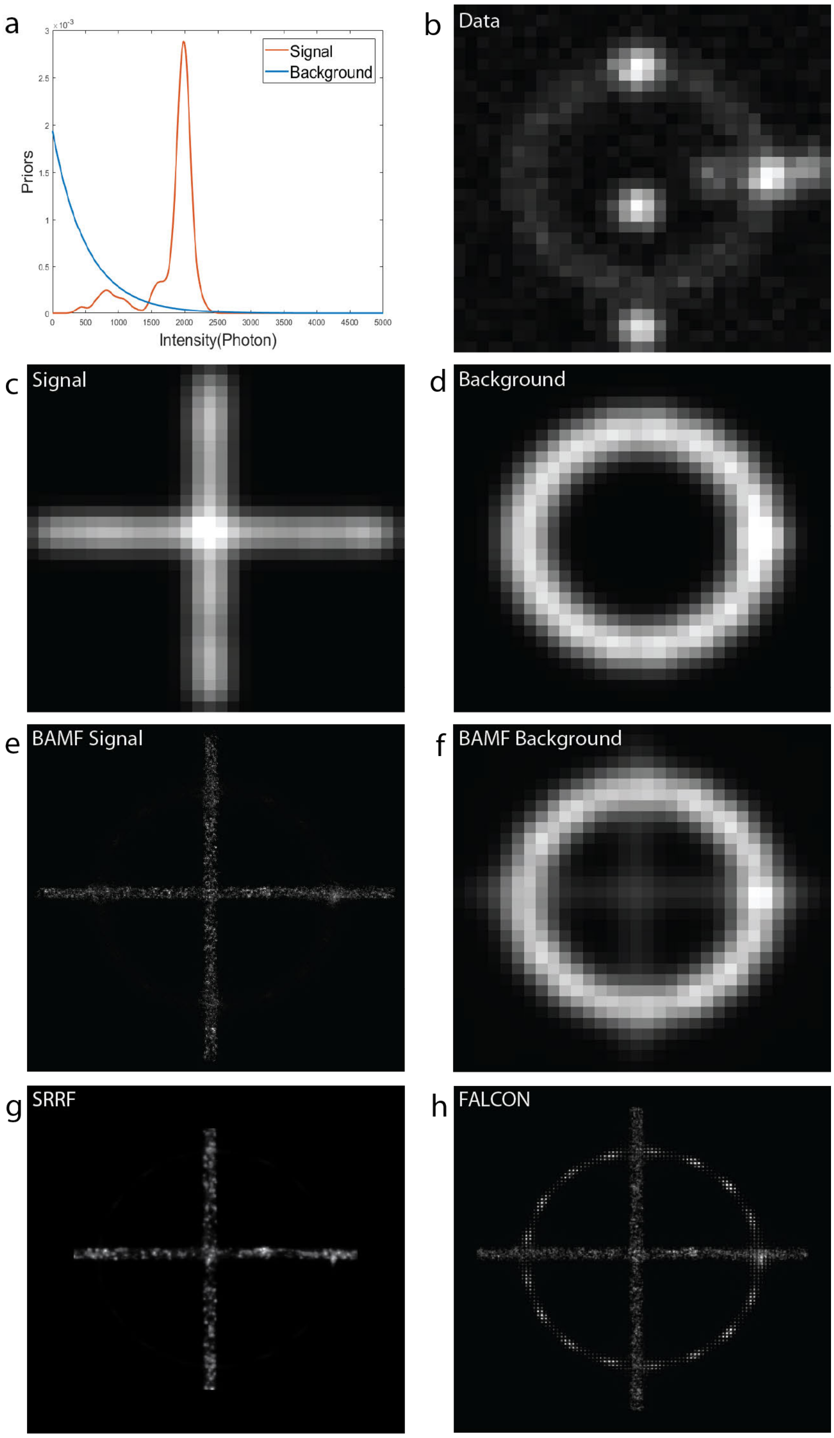

Supplementary Figure 5: Separation of the signal from the structured background. (a) Plots of the priors used for signal and background emitters. (b) One frame of the simulated data. (c) Noise-free simulated signal. (d) Noise-free simulated structured background. (e) Super-resolved reconstruction from BAMF. (f) The structured background reconstructed using the background emitters from BAMF. (g) Super-resolved reconstructions by SRRF. (h) Super-resolved reconstructions by FALCON. Each image was scaled independently.

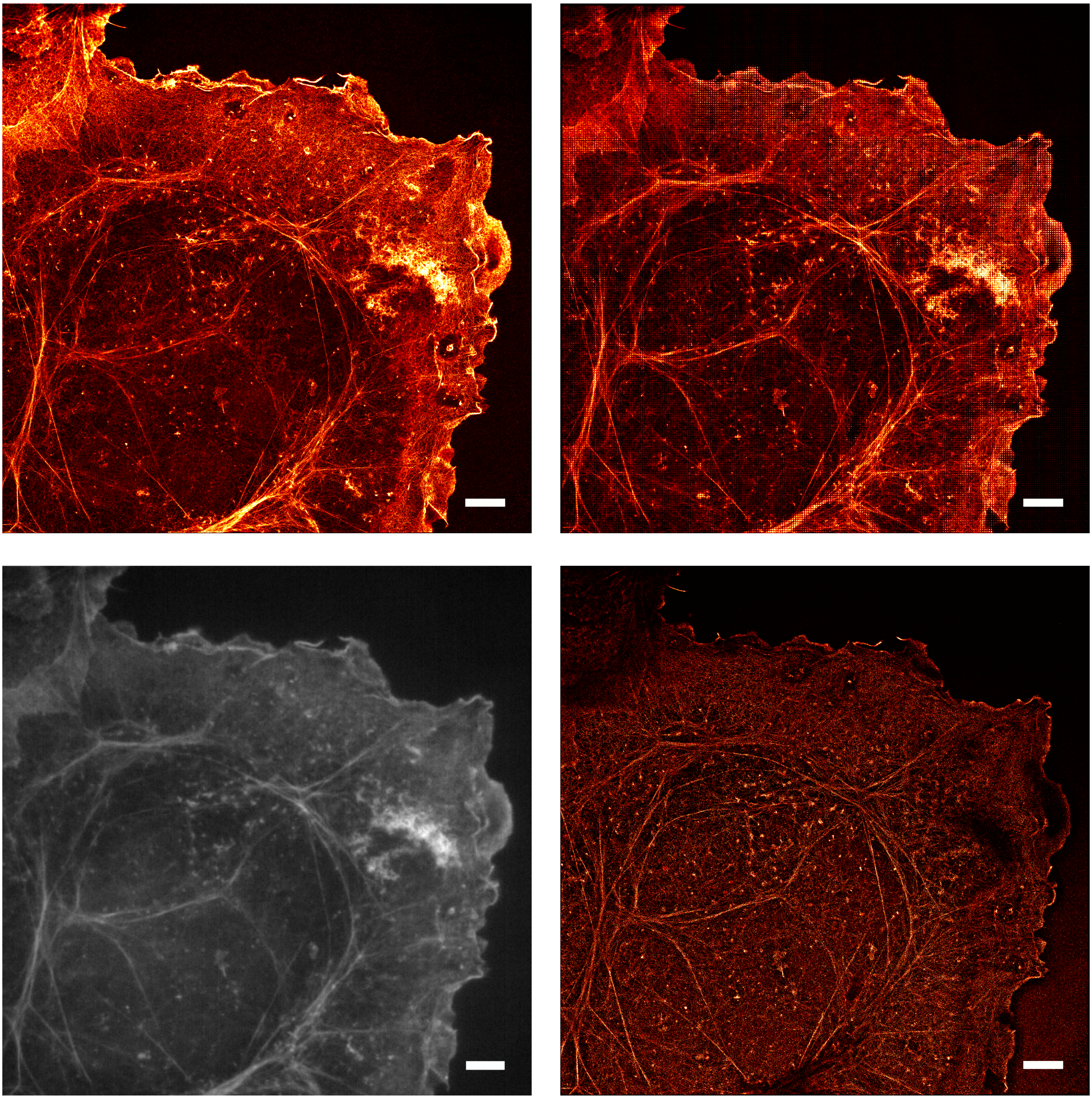

Supplementary Figure 6: Actin filaments in Cos7 cells using DNA-PAINT. Reconstructions from BAMF, FALCON and the single-emitter fitting code for DNA-paint data. (top left) Posterior image from BAMF, (top right) reconstruction from FALCON, (bottom left) wide field image, (bottom right) reconstruction from the single-emitter fitting code. The scale bars are 5 μm.

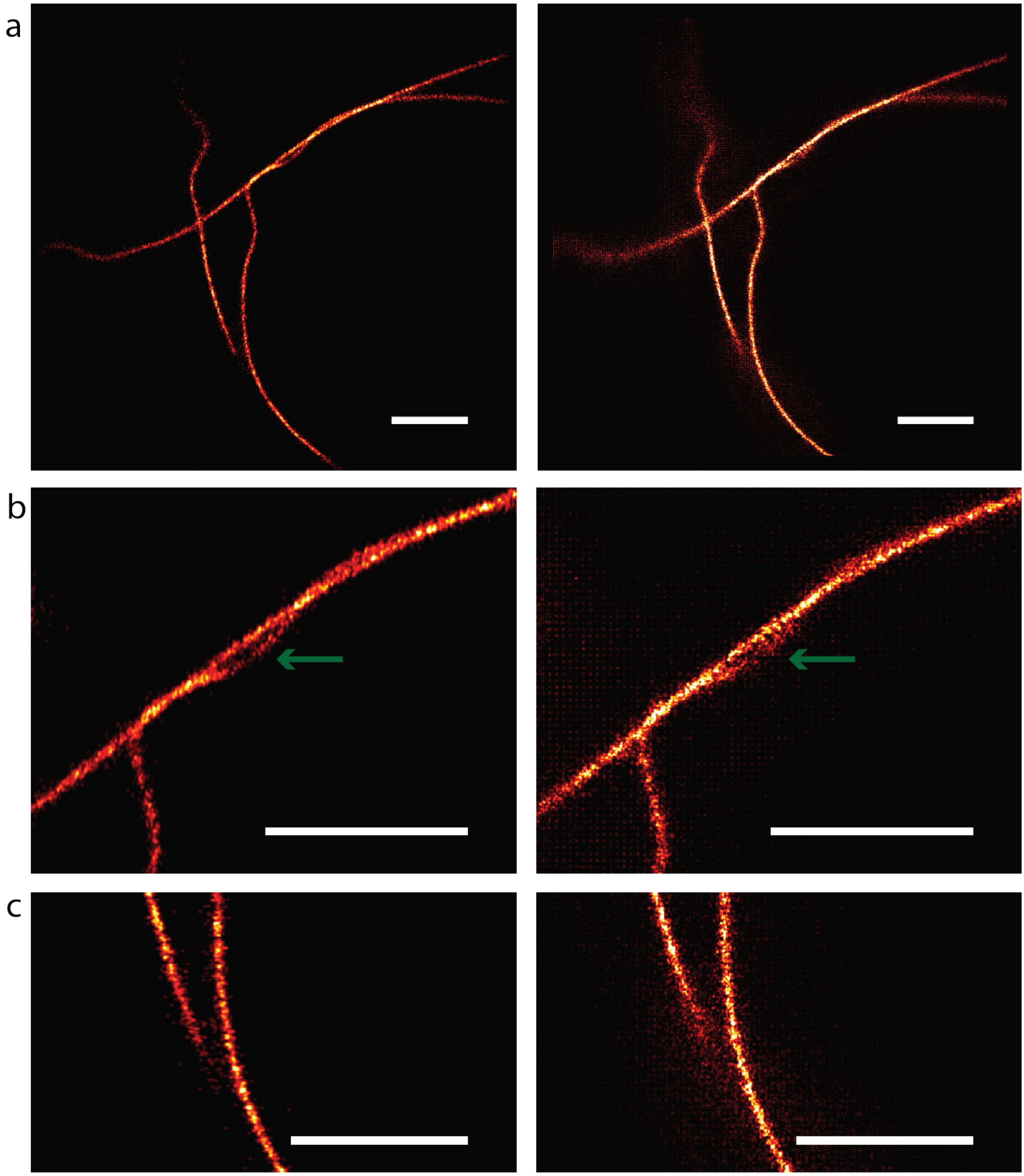

Supplementary Figure 7: The dense, low signal to noise simulated microtubules data (MT4.N2.HD (2D)) from the SMLM challenge website. The first and second columns show the reconstructions from BAMF and FALCON, in turn. Row (b) shows a zoomed in region from the reconstructions. The green arrows point to slightly separated microtubules. (c) Right, FALCON models structured background with signal emitters. Left, BAMF does not include background emitters in the final reconstruction. Scale bars are 1 μm.

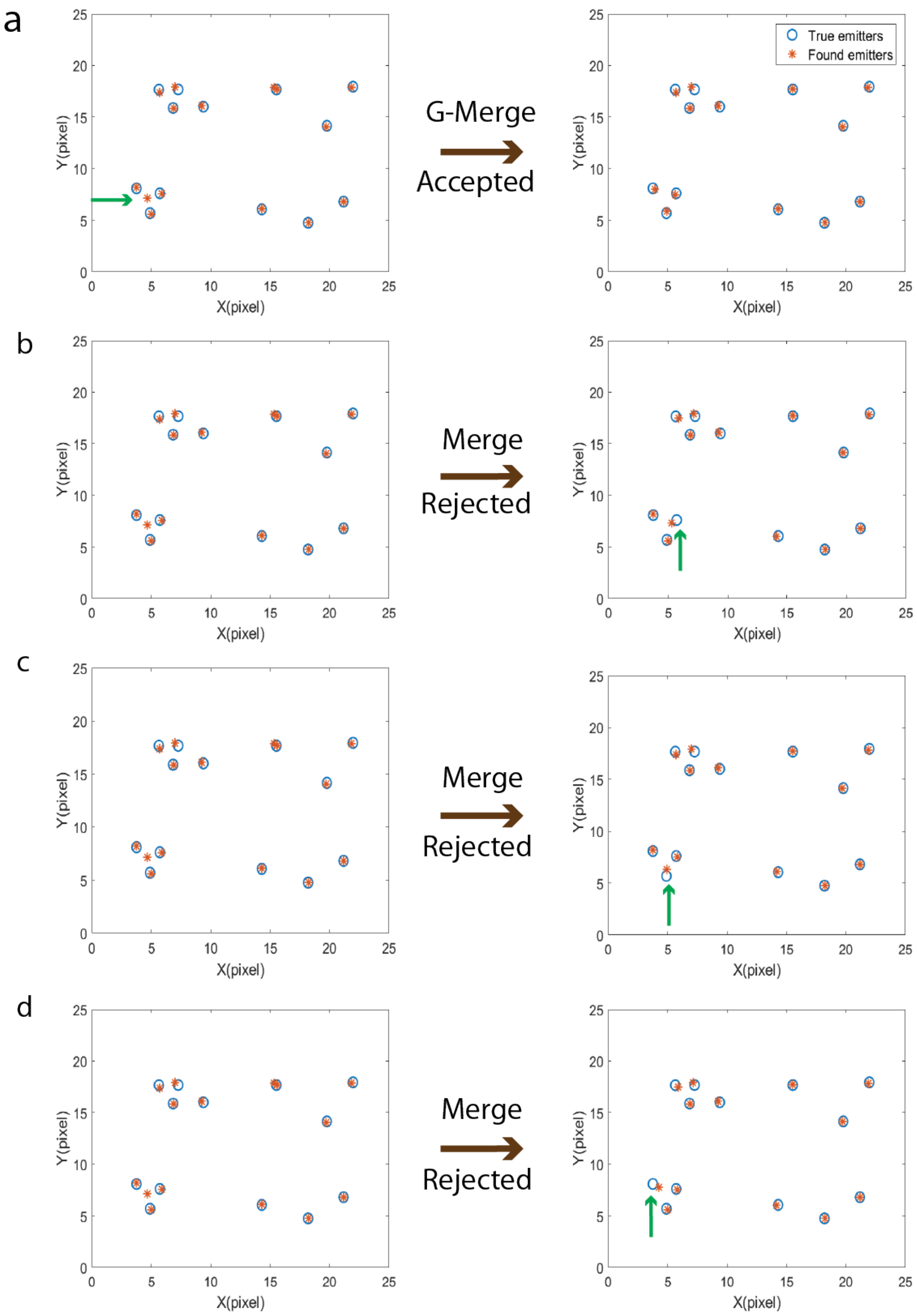

Supplementary Figure 8: Generalized merge example. The blue circles show the true emitters and the red stars show the found emitters. The arrow on the top left plot shows an extra emitter placed in the middle of three emitters. (b) , (c) and (d) describe proposed merges of the middle emitter with each one of the three neighboring emitters. (b), (c) and (d) were rejected jumps. (a) shows a generalized merge that was accepted and the chain escaped from the local maximum. In the generalized merge jump, the photons from the middle emitter were distributed among the three adjacent emitters.

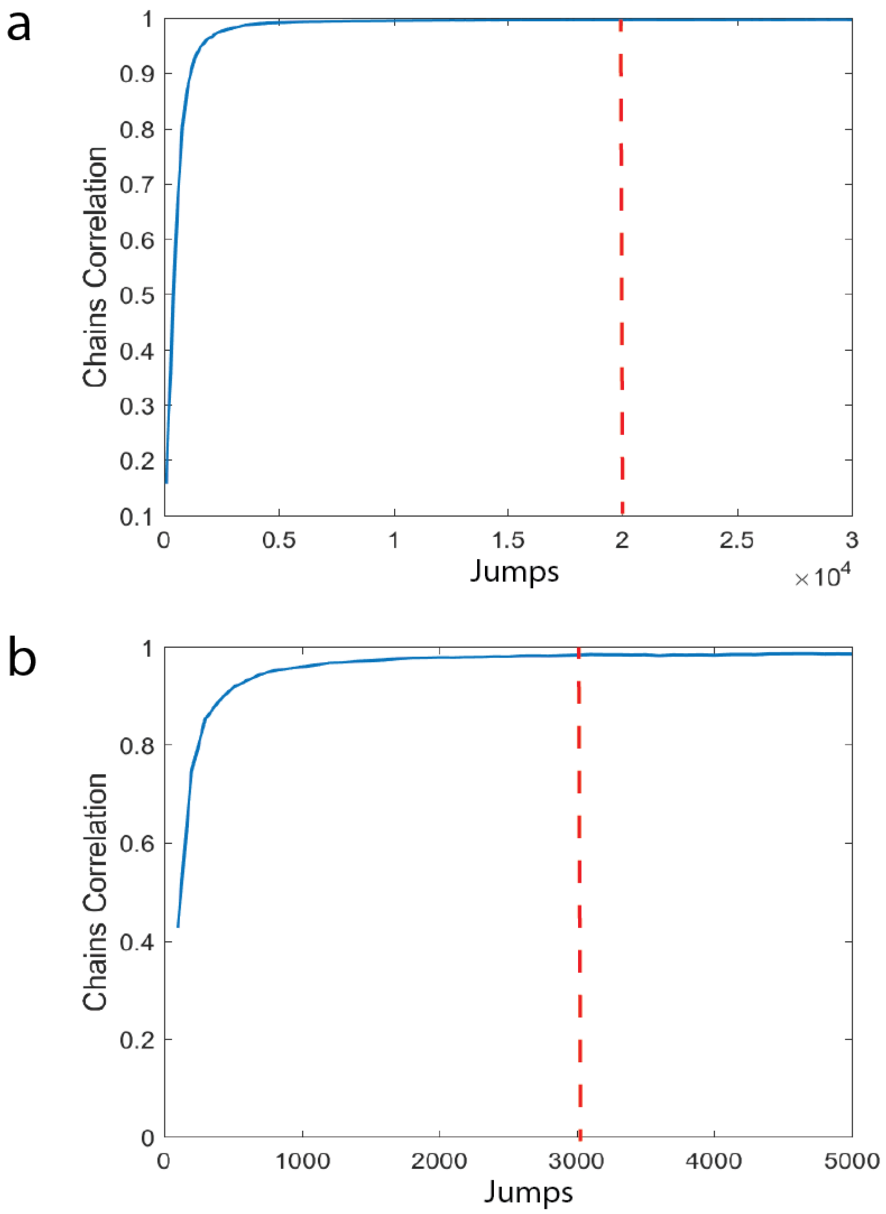

Supplementary Figure 9: Chains correlation and convergence. Plots show the average correlations for chains from two independent runs over the same 20 different regions of data. We uses 30,000 jumps to process the data set used for JAC and the two nearby emitters. (a) shows the chain correlations for the data set used to generate JAC. For the analyses of other data sets, 5,000 jumps were used. (b) shows the chain correlation for STORM data. The red dashed lines separate the burn-in and post-burn-in portions of the chains.

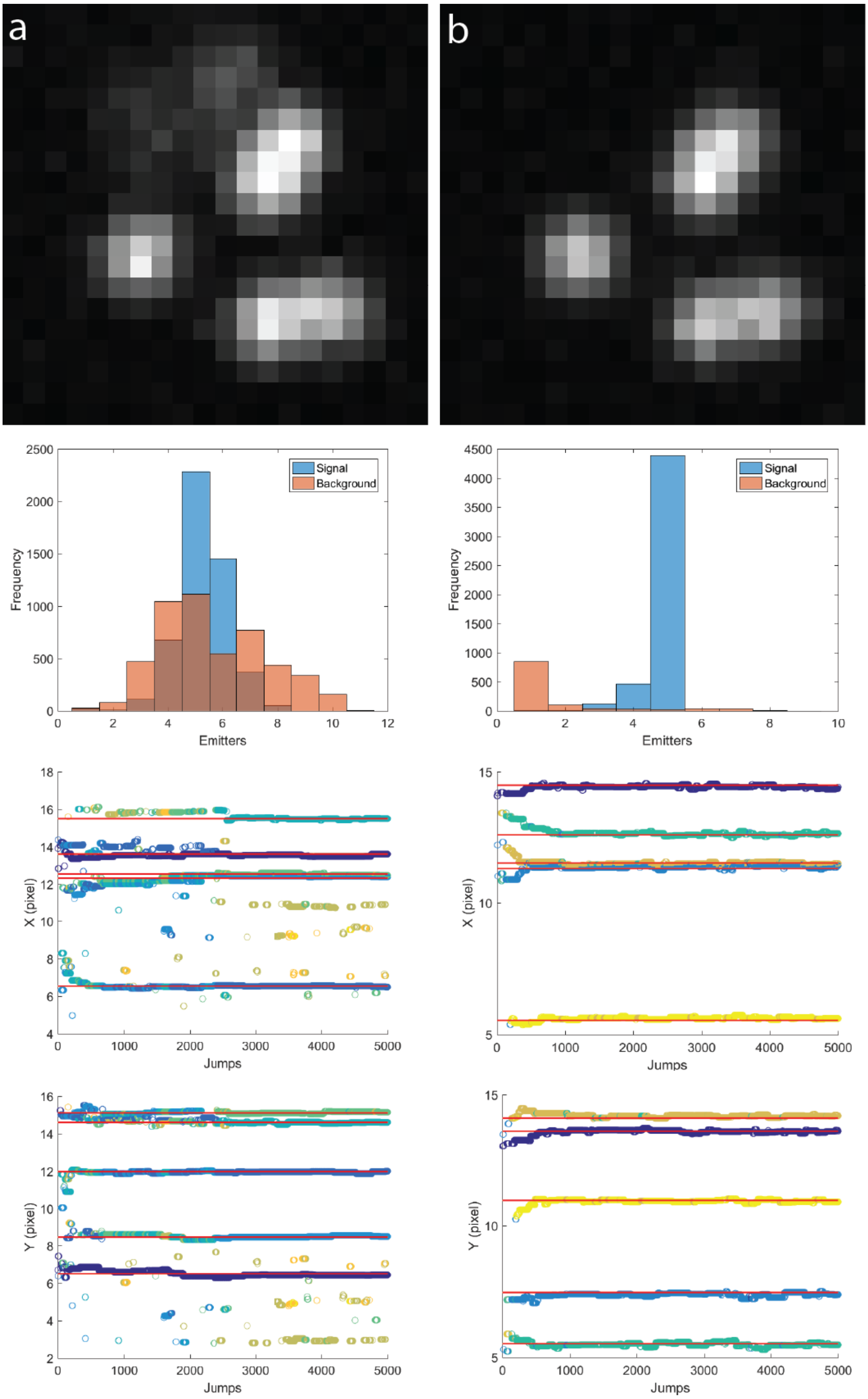

Supplementary Figure 10: Chain mixing and convergence. Column a: chain mixing and convergence in the presence of structured background. Column b: chain mixing and convergence in the absence of structured background. First row: the simulated data of 5 signal emitters in the presence and absence of structured background. Second row: the histogram of the number of detected emitters. Third row: plots of x-locations vs number of jumps. The red lines represent the true locations. Fourth row: plots of y-locations vs number of jumps. The outliers in the plots in column a are due to the classification of the structured background as signal emitters and show the classification uncertainty. Different colors show orders of emitters in the chain.

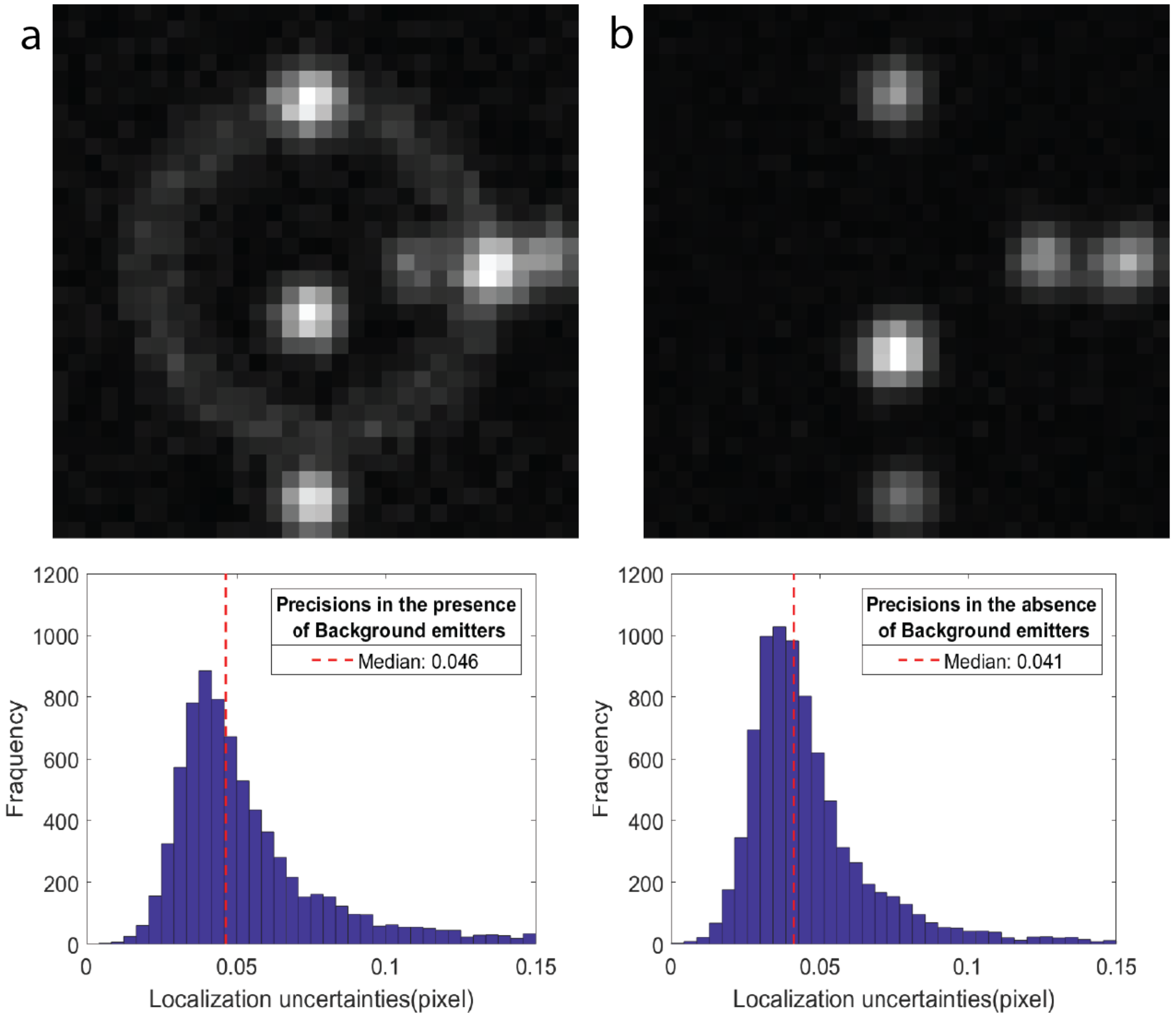

Supplementary Figure 11: The localization precisions in the presence and absence of structured background. Column (a) shows a sample frame of simulated data with structured background and the resulting precisions from BAMF. Column (b) displays a sample frame of the same simulated data in the absence of structured background and the output precisions from BAMF.

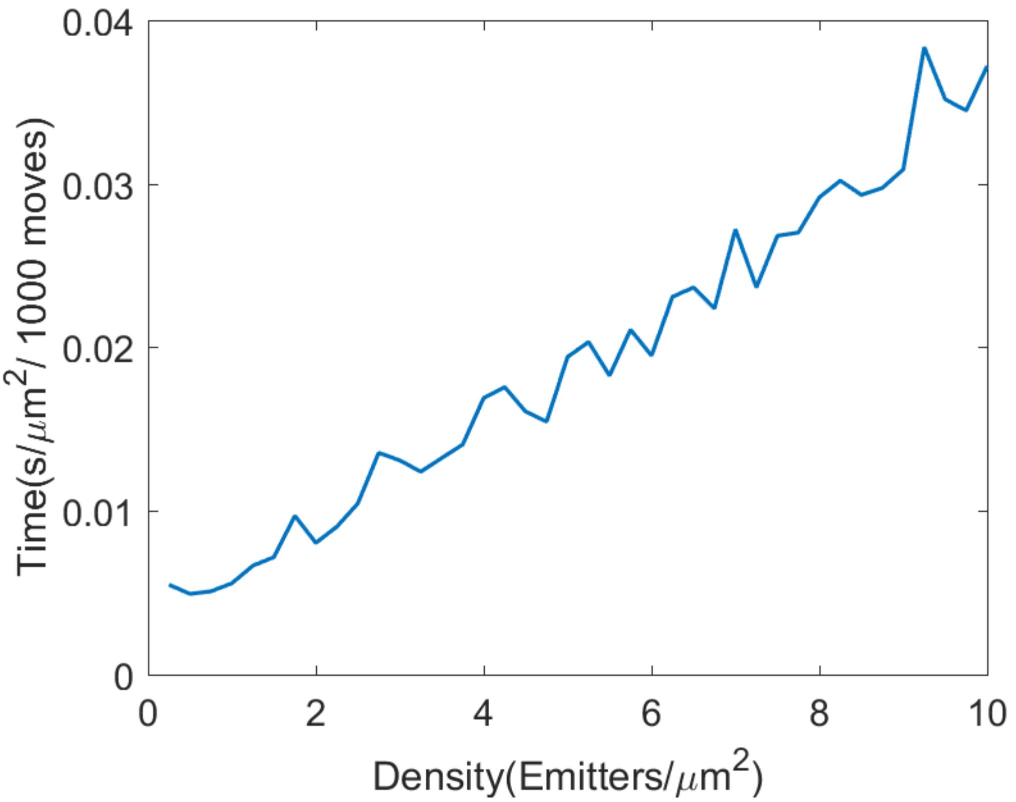

Supplementary Figure 12: The computational cost versus the density of emitters. The computational cost increases almost linearly with the density of emitters.

1 Supplementary Note 1: Reversible Jump Markov Chain Monte Carlo

Markov Chain Monte Carlo (MCMC) methods are restricted to problems where the number of the parameters is fixed [17,18]. Reversible Jump Markov Chain Monte Carlo (RJMCMC) is an extension of MCMC, in which jumps between parameter spaces with different numbers of parameters are permitted and hence makes inferences about the number of parameters as well as the parameters themselves [15,16]. In other words, the number of the parameters is one of the unknowns in RJMCMC. For the multiple-emitter fitting problem that we will address here, the number of parameters and the parameters correspond to the number of emitters, N, their locations, x, y, intensities, I, an offset background, b, and two slopes of the offset plane along the X and Y axes, a_x, a_y (Table 1 & **Methods; PSF model and likelihood**). There are ten different types of jumps in BAMF (**Supplementary Fig. 1**). The conversion jump classifies the found emitters as either background or signal. There are three different types of inside model moves (single-emitter move, group move, background move). These moves make inferences about the positions and intensities of the emitters and do not hop between different models. The other six jumps allow removing or adding emitters to the models and jumping to a new model with either one less or one more emitter. The first jump is split, which tests if there is possibility for an existing emitter to split into two adjacent emitters. Merge is the inverse of split. It considers the chance of two close emitters to really be a single emitter. Generalized split investigates the possibility of N nearby emitters to actually be $N + 1$ emitters. Generalized merge calculates the probability of $N + 1$ adjacent emitters to consolidate into N emitters. The generalized split and generalized merge jumps are especially useful in dense super-resolution data (**Supplementary Fig. 8**). Birth explores different parts of the data to see if a new emitter can be added to the current model. Death is the opposite of birth and examines the feasibility of eliminating one of the existing emitters from the current model.

1.1 Jump Types

BAMF defines three main types of jumps to sample the posterior of the system in the core RJMCMC algorithm. The first type of jump is the within model jump, which optimizes the parameters while not changing the number of the parameters or the number of the emitters. The second type of jump are those that allow the chain to move between different parameter spaces, varying the number of emitters for the given data. The third type of jump is between the background and signal states, which sorts the signal emitters out from the background emitters.

1.1.1 Proposing a Jump

In the following, we explain how these jumps are proposed and how we accept or reject them. First, the chain is initialized to an arbitrary state, containing an arbitrary number of emitters with random locations and intensities. We then pick a random number from a uniform distribution over the interval $[0, 1]$, and based on this random number propose a jump. The occurrence probability of each jump is given by the user, which we call $P_J, P_S, P_M, P_{GS}, P_{GM}, P_B, P_D$ and P_C respectively for the probabilities of proposing a within model jump, a split, a merge, a generalized split, a generalized merge, a birth, a death or a conversion (for instance $P_J = P_S = P_M = P_{GS} = P_{GM} = P_B = P_D = P_C = 1/8$). Note that we have

$$P_J + P_S + P_M + P_{GS} + P_{GM} + P_B + P_D + P_C = 1. \tag{1}$$

A random number *rand* is taken from the interval $[0, 1]$. The jump n (where the jumps above are labeled sequentially) is then proposed if

$$\sum_{i=1}^{n-1} P_i \leq \text{rand} < \sum_{i=1}^{n} P_i \tag{2}$$

After proposing the jump, we calculate the model and the acceptance probability. Next, another random number is picked in the interval $[0, 1]$. The jump will be accepted if the acceptance probability is larger than this random number; otherwise it will be rejected.

1.1.2 Within Model Moves

A within model move does not change the number of the emitters or the number of parameters. It only changes the parameters related to the background, positions and intensities of the existing emitters. The move sizes for different parameters in BAMF algorithm are taken from normal distributions. The widths of these normal distributions determine the size of the moves as well as the acceptance rates of the within model moves [26].

Single-emitter Move

An emitter or a set of neighboring emitters is taken from the list of current emitters at random. The new parameters, which we show by prime, are given by

$$\vec{x'} = \vec{x} + \Delta\vec{x}, \quad \vec{y'} = \vec{y} + \Delta\vec{y}, \quad \vec{I'} = \vec{I} + \Delta\vec{I} \tag{3}$$

where $\Delta x, \Delta y$ and ΔI are taken from normal distributions with centers at the origin and the widths are provided by the user for determining the jump sizes. The acceptance probability of a move from θ to θ' for in-model updates is given by

$$P = \min\left\{1, \frac{p(\theta'|D)}{p(\theta|D)}\right\} \tag{4}$$

where $P(\theta'|D)$ and $P(\theta|D)$ denote the posterior of the proposed parameters and the current parameters given in **Methods; PSF model and likelihood**. The ratio in (4) can be expanded as

$$\frac{P(\theta'|D)}{P(\theta|D)} = \left(\prod_k e^{\lambda_k - \lambda'_k} \left(\frac{\lambda'_k(N)}{\lambda_k(N)}\right)^{D_k}\right) \left(\prod_{n=1}^{N} \frac{P(I'_n)}{P(I_n)}\right) \tag{5}$$

where k and n count the pixels and emitters, respectively. The first parenthesized expression is the ratio of the likelihoods, while the second one is from the prior intensity in which the position priors are canceled. Note that the evidences cancel, simplifying the calculations tremendously because obtaining the evidence involves computing very complicated integrals, **Methods; Priors and posterior**.

Group Move

In a group move, a group of neighboring emitters is found, and then we take a random subset of that group. Next, the intensities of the chosen emitters are redistributed among themselves, and we move the emitters so that their center of mass is conserved. In other words, we keep the center of mass and the number of photons conserved in this move. This jump is especially worthwhile for denser data sets as it helps to escape from local maximums in the dense regions where a single-emitter move fails. The acceptance probability is calculated utilizing (4,5).

Background Move

A background move does not deal with any of the emitters' parameters. Instead, it attempts to optimize the offset background and its slopes along the X and Y axes. The jumps are taken from normal distributions where the step size in offset background is provided by the user.

$$b' = b + \Delta b, \quad a'_x = a_x + \Delta a_x, \quad a'_y = a_y + \Delta a_y \tag{6}$$

The acceptance probability is computed as before.

1.1.3 Split and Merge

Split and merge are two complementary reversible mechanisms that allow for exploring the possibility of a different number of emitters in a local area. Basically, in each step single emitters are allowed to divide into two, or two neighboring emitters are allowed to combine. The new/revised emitters produced by split/merge are classified as either signal or background based on the new intensities.

Split

On proposing a split, an emitter is chosen at random. After splitting this emitter into two emitters, we will have two sets of parameters rather than one, and thus we choose to calculate these new sets of parameters based on conservation principles. Our rules for the parameters of the new emitters are that the total intensity (zero moment) and center of mass (first moment) are conserved.

$$\begin{aligned} I_j &= I_{j^1} + I_{j^2} \\ I_j x_j &= I_{j^1} x_{j^1} + I_{j^2} x_{j^2} \\ I_j y_j &= I_{j^1} y_{j^1} + I_{j^2} y_{j^2} \end{aligned} \tag{7}$$

where the subscripts j, j^1 and j^2 stand for the randomly chosen emitter and the two new emitters after splitting. There are still degrees of freedom in how to do this, so we generate a random vector $\vec{u}$ whose elements are used to calculate the specific parameters. Select u_1 from Beta(1,1), and u_2 and u_3 from $N(0, \sigma^2_{\text{PSF}})$. Solving for the new parameters

$$\begin{aligned} I_{j^1} &= I_j u_1 \\ I_{j^2} &= I_j(1-u_1) \\ x_{j^1} &= x_j + u_2 \\ y_{j^1} &= y_j + u_3 \\ x_{j^2} &= x_j - \frac{u_1 u_2}{1-u_1} \\ y_{j^2} &= y_j - \frac{u_1 u_3}{1-u_1} \end{aligned} \tag{8}$$

The acceptance probability for a cross-dimensional jump in RJMCMC is [15,16]

$$P = \min\{1, A\} \tag{9}$$

where

$$A = \frac{P(\theta'|D) r_m(\theta')}{P(\theta|D) r_m(\theta) q(u)} \left| \frac{\partial(\theta')}{\partial(\theta, u)} \right| \tag{10}$$

$P(\theta'|D)$ is the posterior; D is data; θ is the current parameter set; θ' means either the deterministic function that calculates the new parameters $\theta'(\theta, u)$ or the result of that calculation; $r_m(\theta)$ is the probability of choosing the move type m when in state θ; and $q(u)$ is the density function of u. The term on the right is the Jacobian for transforming variables from (θ, u) to θ'. The ratio $\frac{P(\theta'|D)}{P(\theta|D)}$ is determined from the details given in Section 1.1, and for split can be written as

$$\frac{P(\theta'|D)}{P(\theta|D)} = \left(\prod_k \frac{L_k(D|\theta')}{L_k(D|\theta)} \right) \left(\frac{\prod_{n=1}^{N+1} P(I'_n)}{\prod_{n=1}^{N} P(I_n)} \right) \frac{1}{(W+2)^2} \left(\frac{\rho W^2}{N+1} \right) \tag{11}$$

where k counts the pixels and n indexes the emitters. In addition, L_k is the likelihood given in **Methods; PSF model and likelihood**, the second parenthesized expression gives the ratio of the intensity priors, the third factor is the ratio of the position priors where W is the width of the given frame in pixels, and the last parenthesized expression is the ratio of the priors for the proposed and current number of emitters. The number of emitters has a Poisson distribution with mean value ρW^2, where ρ is the density of emitters and N is the current number of emitters. The ratio $\frac{r_m(x')}{r_m(x)}$ is $\frac{P_M}{P_S}$, because the probabilities for proposing a split and merge are given by P_S and P_M. $q(u)$ is the probability density of u, that is, $p(u_1)p(u_2)p(u_3)$, where these probabilities are calculated from the PDFs used to generate $\vec{u}$. The term $\left| \frac{\partial(\theta')}{\partial(\theta,u)} \right|$ is written out here. Only the values of θ' related to the split have been affected, so all other elements of this $3(N+1) \times 3(N+1)$ matrix are diagonal except those relating to the 6 changed elements of θ'. Putting these elements in the order listed above, $\theta' = (..., I_{j^1}, x_{j^1}, x_{j^2}, I_{j^2}, y_{j^1}, y_{j^2})$ and $(\theta, u) = (..., I_j, u_1, x_j, u_2, y_j, u_3)$. The Jacobian matrix is

$$\frac{\partial(\theta')}{\partial(\theta, u)} = \begin{pmatrix} 1-u_1 & 0 & 0 & -I_j & 0 & 0 \\ u_1 & 0 & 0 & I_j & 0 & 0 \\ 0 & 1 & 0 & \frac{-u_2}{1-u_1} + \frac{-u_2 u_1}{(1-u_1)^2} & \frac{-u_1}{1-u_1} & 0 \\ 0 & 1 & 0 & 0 & 1 & 0 \\ 0 & 0 & 1 & \frac{-u_3}{1-u_1} + \frac{-u_3 u_1}{(1-u_1)^2} & 0 & \frac{-u_1}{1-u_1} \\ 0 & 0 & 1 & 0 & 0 & 1 \end{pmatrix} \tag{12}$$

and the determinant is therefore

$$\left| \frac{\partial(\theta')}{\partial(\theta, u)} \right| = \frac{I_j}{(1-u_1)^2} \tag{13}$$

The complete expression for A is

$$\begin{aligned} A &= \left(\prod_k \frac{e^{-\lambda_k(N+1)} \lambda_k(N+1)^{D_k}}{e^{-\lambda_k(N)} \lambda_k(N)^{D_k}} \right) \frac{1}{(W+2\sigma)^2} \frac{\prod_{n=1}^{N+1} P(I'_n)}{\prod_{n=1}^{N} P(I_n)} \left(\frac{\rho W^2}{N+1} \right) \\ &\times \frac{P_M}{P_S} (\text{Beta}(u_1,1,1) N(u_2, 0, \sigma^2_{\text{PSF}}) N(u_3, 0, \sigma^2_{\text{PSF}}))^{-1} \frac{I_j}{(1-u_1)^2} \end{aligned} \tag{14}$$

Merge

After proposing a merge, we randomly pick an emitter to combine with one of its neighbors. If the neighboring emitter of the picked emitter is further than $2\sigma_{\text{PSF}}$, we reject the proposed merge, otherwise we calculate the acceptance probability given by

$$P = \min\{1, A^{-1}\} \tag{15}$$

where u_1, u_2, u_3 are deterministically calculated from (8) and A is given by (14).

1.1.4 Generalized Split and Merge

Split and merge allow movement from one emitter to two emitters and vice versa. In dense data sets, a generalized version of those jumps proved to be pivotal for efficient mixing of the chain and escapes from local maxima, where they permit moving from N emitters to $N+1$ emitters and vice versa, **Supplementary Figure 8**. Therefore, we can explore the probability of N nearby emitters to be $N+1$ emitters and the opposite. The group of N nearby emitters are selected by picking an emitter at random and then finding the emitters that are closer than $2\sigma_{\text{PSF}}$ to it. The new/revised emitters are classified as either signal or background based on the new intensities.

Generalized Split

A random group of N adjacent emitters is picked. A new emitter is then formed by taking a few photons from each of the picked emitters. This takes us to a model with one more emitter. The parameters of the new emitters are calculated so that the number of photons and the center of mass are conserved.

$$\begin{aligned} \sum_{i=1}^{N} I_i &= \sum_{j=1}^{N+1} I'_j \\ \sum_{i=1}^{N} I_i x_i &= \sum_{j=1}^{N+1} I'_j x'_j \\ \sum_{i=1}^{N} I_i y_i &= \sum_{j=1}^{N+1} I'_j y'_j \end{aligned} \tag{16}$$

where prime indicates the parameters of the proposed emitters. The primed parameters are $3(N+1)$ unknowns. The three equations given in (16) can be used to reduce the degrees of freedom, however, this still leaves $3N$ degrees of freedom for the primed parameters. To eliminate these freedoms, we propose the following equations

$$\begin{aligned} I'_i &= (1-u_1)I_i \\ x'_i &= \frac{x_i - u_1\left(\frac{1}{N}\sum_{i=1}^{N} x_i + u_2\right)}{1-u_1} \\ y'_i &= \frac{y_i - u_1\left(\frac{1}{N}\sum_{i=1}^{N} y_i + u_3\right)}{1-u_1} \end{aligned} \tag{17}$$

where $i = 1, ..., N$. The first equation above says that the percentage of photons taken from each existing emitter is equal. The second and third equations in (17) are derived by assuming that the new emitter lies in the vicinity of the center of mass of the existing emitters. However, this still leaves 3 degrees of freedom to be set. The random variables $u_1 \sim \text{beta}, u_2 \sim \text{normal}, u_3 \sim \text{normal}$ are then introduced to satisfy them. Using these random variables and (16,17), we can deterministically solve for the parameters of the new emitter

$$\begin{aligned} I'_{N+1} &= u_1 \sum_{i=1}^{N} I_i \\ x'_{N+1} &= \frac{1}{N}\sum_{i=1}^{N} x_i + u_2 \\ y'_{N+1} &= \frac{1}{N}\sum_{i=1}^{N} y_i + u_3 \end{aligned} \tag{18}$$

The acceptance probability for generalized split is given by

$$P = \min\{1, A\} \tag{19}$$

where A is given by (10) and the Jacobian can be calculated as follows

$$\frac{\partial(\theta')}{\partial(\theta,u)} = \begin{bmatrix} \frac{\partial I'_1}{\partial I_1} & \cdots & \frac{\partial I'_1}{\partial I_N} & \frac{\partial I'_1}{\partial u_1} & \frac{\partial I'_1}{\partial x_1} & \cdots & \frac{\partial I'_1}{\partial x_N} & \frac{\partial I'_1}{\partial u_2} & \frac{\partial I'_1}{\partial y_1} & \cdots & \frac{\partial I'_1}{\partial y_N} & \frac{\partial I'_1}{\partial u_3} \\ \vdots & \ddots & \vdots & \vdots & \vdots & \ddots & \vdots & \vdots & \vdots & \ddots & \vdots & \vdots \\ \frac{\partial I'_N}{\partial I_1} & \cdots & \frac{\partial I'_N}{\partial I_N} & \frac{\partial I'_N}{\partial u_1} & \frac{\partial I'_N}{\partial x_1} & \cdots & \frac{\partial I'_N}{\partial x_N} & \frac{\partial I'_N}{\partial u_2} & \frac{\partial I'_N}{\partial y_1} & \cdots & \frac{\partial I'_N}{\partial y_N} & \frac{\partial I'_N}{\partial u_3} \\ \frac{\partial I'_{N+1}}{\partial I_1} & \cdots & \frac{\partial I'_{N+1}}{\partial I_N} & \frac{\partial I'_{N+1}}{\partial u_1} & \frac{\partial I'_{N+1}}{\partial x_1} & \cdots & \frac{\partial I'_{N+1}}{\partial x_N} & \frac{\partial I'_{N+1}}{\partial u_2} & \frac{\partial I'_{N+1}}{\partial y_1} & \cdots & \frac{\partial I'_{N+1}}{\partial y_N} & \frac{\partial I'_{N+1}}{\partial u_3} \\ \frac{\partial x'_1}{\partial I_1} & \cdots & \frac{\partial x'_1}{\partial I_N} & \frac{\partial x'_1}{\partial u_1} & \frac{\partial x'_1}{\partial x_1} & \cdots & \frac{\partial x'_1}{\partial x_N} & \frac{\partial x'_1}{\partial u_2} & \frac{\partial x'_1}{\partial y_1} & \cdots & \frac{\partial x'_1}{\partial y_N} & \frac{\partial x'_1}{\partial u_3} \\ \vdots & \ddots & \vdots & \vdots & \vdots & \ddots & \vdots & \vdots & \vdots & \ddots & \vdots & \vdots \\ \frac{\partial x'_N}{\partial I_1} & \cdots & \frac{\partial x'_N}{\partial I_N} & \frac{\partial x'_N}{\partial u_1} & \frac{\partial x'_N}{\partial x_1} & \cdots & \frac{\partial x'_N}{\partial x_N} & \frac{\partial x'_N}{\partial u_2} & \frac{\partial x'_N}{\partial y_1} & \cdots & \frac{\partial x'_N}{\partial y_N} & \frac{\partial x'_N}{\partial u_3} \\ \frac{\partial x'_{N+1}}{\partial I_1} & \cdots & \frac{\partial x'_{N+1}}{\partial I_N} & \frac{\partial x'_{N+1}}{\partial u_1} & \frac{\partial x'_{N+1}}{\partial x_1} & \cdots & \frac{\partial x'_{N+1}}{\partial x_N} & \frac{\partial x'_{N+1}}{\partial u_2} & \frac{\partial x'_{N+1}}{\partial y_1} & \cdots & \frac{\partial x'_{N+1}}{\partial y_N} & \frac{\partial x'_{N+1}}{\partial u_3} \\ \frac{\partial y'_1}{\partial I_1} & \cdots & \frac{\partial y'_1}{\partial I_N} & \frac{\partial y'_1}{\partial u_1} & \frac{\partial y'_1}{\partial x_1} & \cdots & \frac{\partial y'_1}{\partial x_N} & \frac{\partial y'_1}{\partial u_2} & \frac{\partial y'_1}{\partial y_1} & \cdots & \frac{\partial y'_1}{\partial y_N} & \frac{\partial y'_1}{\partial u_3} \\ \vdots & \ddots & \vdots & \vdots & \vdots & \ddots & \vdots & \vdots & \vdots & \ddots & \vdots & \vdots \\ \frac{\partial y'_N}{\partial I_1} & \cdots & \frac{\partial y'_N}{\partial I_N} & \frac{\partial y'_N}{\partial u_1} & \frac{\partial y'_N}{\partial x_1} & \cdots & \frac{\partial y'_N}{\partial x_N} & \frac{\partial y'_N}{\partial u_2} & \frac{\partial y'_N}{\partial y_1} & \cdots & \frac{\partial y'_N}{\partial y_N} & \frac{\partial y'_N}{\partial u_3} \\ \frac{\partial y'_{N+1}}{\partial I_1} & \cdots & \frac{\partial y'_{N+1}}{\partial I_N} & \frac{\partial y'_{N+1}}{\partial u_1} & \frac{\partial y'_{N+1}}{\partial x_1} & \cdots & \frac{\partial y'_{N+1}}{\partial x_N} & \frac{\partial y'_{N+1}}{\partial u_2} & \frac{\partial y'_{N+1}}{\partial y_1} & \cdots & \frac{\partial y'_{N+1}}{\partial y_N} & \frac{\partial y'_{N+1}}{\partial u_3} \end{bmatrix} \tag{20}$$

$$\left|\frac{\partial(\theta')}{\partial(\theta,u)}\right| = \frac{\sum_{i=1}^{N} I_i}{(1-u_1)^{N+1}} \tag{21}$$

Note that the above equations can be reduced to the equations in the split section when $N = 1$.

Generalized Merge

An emitter is chosen at random and then we pick N random emitters closer that $2\sigma_{\text{PSF}}$. The picked emitter (emitter $N+1$) is annihilated and its photons are distributed among the set of the neighboring emitters. Note that we jump from $N+1$ emitters to N emitters. The emitters' parameters and the random u's may be calculated in a deterministic way

$$\begin{aligned} u_1 &= \frac{I_{N+1}}{\sum_{i=1}^{N+1} I_i} \\ I'_i &= \frac{I_i}{1-u_1} \\ x'_i &= u_1 x_{N+1} + (1-u_1)x_i \\ y'_i &= u_1 y_{N+1} + (1-u_1)y_i \\ u_2 &= x_{N+1} - \frac{1}{N}\sum_{i=1}^{N} x'_i \\ u_3 &= y_{N+1} - \frac{1}{N}\sum_{i=1}^{N} y'_i \end{aligned} \tag{22}$$

where parameters with the prime represent the parameters associated with the proposed model (one less emitter). The acceptance probability for this jump is given by

$$P = \min\{1, A^{-1}\} \tag{23}$$

where A is described in the previous section.

1.1.5 Birth and Death

Birth and death are complementary reversible processes. They allow addition and subtraction of emitters globally. Birth inspects the data to find the spots where an emitter might exist. Death removes one of the existing emitters at random to examine the probability of the model with one less emitter.

Birth

To find a suitable spot for the new emitter, we subtract the current model from the data to obtain the residuum image. The residuum image can be interpreted as a probability distribution where pixels with higher values indicate higher probability for a missing emitter. We pick a location at random from this probability distribution for the new emitter. An alternative approach would be proposing a random location for the new emitter across the image where all the pixels have the same probability; however most of the proposed births in this procedure would be rejected. The residuum image scheme facilitates spotting the missing emitters and hence the chain converges faster. The intensity of the new emitter is chosen from the intensity prior at random and therefore $q(u)$ in (10) contains the intensity prior, which cancels the intensity prior from the posterior, and so the intensity prior is not involved in the calculations. The acceptance probability is given by (9), where

$$A = \left(\prod_k \frac{e^{-\lambda_k(N+1)} \lambda_k(N+1)^{D_k}}{e^{-\lambda_k(N)} \lambda_k(N)^{D_k}} \right) \frac{1}{P_{\text{res}}(W+2\sigma)^2} \left(\frac{\rho W^2}{N+1} \right) \frac{P_D}{P_B} \tag{24}$$

Here, the first factor is the likelihood ratio, the second factor is the location prior ratio with P_{res} standing for the residuum image distribution, the third factor is the prior ratio for the number of emitters, and the last factor represents the ratio of the probabilities to propose a death or a birth. The new emitter is randomly classified as either signal or background.

Death

Proposing a death, we pick one of the existing emitters at random and remove it from the model. This is the opposite of birth and its acceptance probability is given by

$$P = \min\{1, A^{-1}\} \tag{25}$$

where

$$A = \left(\prod_k \frac{e^{-\lambda_k(N+1)} \lambda_k(N+1)^{D_k}}{e^{-\lambda_k(N)} \lambda_k(N)^{D_k}} \right) \left(\frac{\rho W^2}{N+1} \right) \frac{P_D}{P_B} \tag{26}$$

The ratio of the position priors is missing here because they exert no preference in picking an emitter.

1.1.6 Conversion

We chose an emitter at random and propose its opposite state. If the emitter is part of the signal, then we will propose it to be a background emitter (Fig. 1f), where the conversion probability is given by

$$P = \min\{1, A\} \tag{27}$$

and

$$A = \frac{P_{\text{Bg}}(I)}{P_{\text{Signal}}(I)} \tag{28}$$

$P_{\text{Signal}}(I)$ and $P_{\text{Bg}}(I)$ represent the signal and background priors (Supplementary Fig. 6a). If the picked emitter is part of the background, then we propose it to be a signal emitter (Fig. 1f) and the acceptance probability can be obtained as follows:

$$P = \min\{1, A^{-1}\} \tag{29}$$

Note that the signal and background emitters contribute to the data and also to the model similarly and the likelihood (**Methods; PSF model and likelihood**) does not depend on the labeling parameter l. Therefore, conversion of an emitter from signal to background or the opposite will not change the likelihood, so they cancel in the ratio (28).

1.2 Chain setup and generation

The above jumps are proposed inside an iterative loop for a user specified number of iterations. Larger regions require more iterations. The chain is built as follows. First, it is initialized to an empty state where there are no emitters and the offset background initial value is taken as the mode of the prior. Next, we propose random states via the available jumps, which are embedded in RJMCMC, and allow exploration of all possible states. Calculating the acceptance probability, which was described above, we either accept or decline the jump. If the jump is accepted, we take it as the next state, otherwise we take the current state as the next one. The beginning part of the chain before its convergence is called the burn-in and will not appear in the final reconstruction. The chain makes many random jumps and attempts to spot the emitters in the given data during the burn-in. The post-burn-in part of the chain is returned by the code and is utilized to generate the final reconstruction.

1.3 Posterior and MAPN outputs

In the post-burn-in chain, the emitters have been detected and the chain has settled to a stationary distribution. There are only small deviations from the real values of the parameters and inter-model jumps may be accepted occasionally, reflecting the uncertainty (Fig. 1e). For each emitter, a blob is returned, where the width is a measure of the uncertainties in the estimated parameters. RJMCMC returns the chain and the posterior image. The posterior image is built by placing a dot on the found emitters' locations in each jump. The posterior images from each subregion are put together to obtain the final chain reconstruction. This chain contains the number of emitters, their positions, intensities, offset background and slopes along the X and Y axes, jump type, likelihood ratio and posterior ratio for each attempted jump. Furthermore, the most repeated model in the chain (MAPN) is extracted, which has a fixed number of emitters. A cloud of localizations made of locations from different states is associated with each emitter in the extracted MAPN chain. k-means clustering, which assumes a fixed number of clusters, is used to combine the locations within each cloud to estimate the localization and uncertainty of the emitter represented by this cloud. This step is necessary because the emitters associated with a given model will have slightly different positions and are ordered arbitrarily in different states, in other words, the emitter IDs are not conserved by inter-model jumps. The results are used to initialize an MCMC chain to further refine the emitter parameters and their uncertainties for the MAPN model by using only within model moves. The MAPN coordinates are then used to generate the MAPN reconstruction from MCMC.

2 Supplementary Note 2: Noise characterization

Two main types of noise in super-resolution data are read-out noise and shot noise. Shot noise comes from the particle nature of photons and can be modeled by a Poisson process. Read-out noise comes from the electronics of the camera and has a Gaussian distribution with the mean value zero. In other words, it is the fluctuations of the detector when there is no signal. For an EMCCD camera, read-out noise can be ignored, however, it has a higher value in the data acquired using a sCMOS camera and cannot be neglected. The entire noise can be modeled as the convolution of the shot noise (Poisson distribution) and read-out noise (Gaussian distribution) [27]

$$P_i(D_i) = N \sum_{q=0}^{\infty} \frac{1}{q!} e^{-u_i} u_i^q \frac{1}{\sqrt{2\pi \mathrm{var}_i}} e^{-\frac{(D-qg_i-o_i)^2}{2\mathrm{var}_i}} \tag{30}$$

where N, D, u, g, o and var are, respectively, the normalization constant, the number of counted photons (Data), the mean photon count, gain, offset and variance with i counting pixels. In the case of EMCCD, the ratio $\frac{\mathrm{var}_i}{g_i^2}$ is small and equation (30) approximates to

$$P(D_i) \approx N' e^{-u_i} u_i^{(D_i-o)/g} \tag{31}$$

where N' is the normalization constant and gain and offset are the same across the EMCCD camera. We used this approximation to obtain the likelihood (**Methods; PSF model and likelihood**). In the case of sCMOS camera, the above approximation is not valid and one should use the analytical expression which can be computationally expensive. An elegant solution to this problem is given in [27], where an additional source of signal is added to the problem with the mean intensity of $\frac{\mathrm{var}_i}{g_i^2}$. Using the fact that the Gaussian distribution asymptotically goes to a Poisson distribution, the read-out noise can also be modeled by a Poisson process. Lastly, the likelihood for the data taken by an sCMOS camera is as follows:

$$L_i(D|\theta) \approx \frac{(\lambda_i(N) + \mathrm{var}_i/g_i)^{D_i} e^{-\lambda_i(N)+\mathrm{var}_i/g_i}}{D_i!} \tag{32}$$

Therefore, adding the term var_i/g_i to the mean number of the photons in the likelihood will take care of the read-out noise in sCMOS cameras.

3 Supplementary Note 3: MATLAB BAMF Library

The RJMCMC algorithm is implemented in C++ as a mex-function which can be called from inside MATLAB. We developed a MATLAB class consisting of several methods and tools along with a user friendly interface (GUI) to facilitate using the algorithm. In this section, a detailed description of the data flow and the methods used at different points of the process are provided. At the end, some of the diagnosis and visualization tools are depicted. Some of the visualization methods can be used to get more insight into RJMCMC and its jumping processes. We place empty parentheses at the end of the method names to make it clear that we are referring to a method.

The main class is called RJ. The input parameters required by the RJMCMC algorithm, such as PSF size, jump sizes, probabilities of proposing different jumps, zoom factor, numerical priors and number of jumps, are all stored in a

structure called RJStruct. To find the intensity and offset background priors, we use a fast single-emitter code which also needs a few input parameters, like mean number of photons, minimum number of photons and minimum p-value, which are stored in a structure called SMAStruct. The post-processing of the data consists of thresholding, frame connection and drift correction. The required parameters for these post-processing steps are stored in structures called Threshold and FrameConnect. Some other inputs like the gain and offset are properties of the RJ-class too. Moreover, some other properties give the user the option to use certain tools or return more outputs, usually for diagnosis, such as using the MATLAB parallel computing toolbox, using a numerical PSF, saving the chain, etc. The structure, ClustInfo, contains the found positions, intensities and backgrounds. JumpStat stores the statistics of all the accepted jumps. PChain and Chain are MATLAB cell arrays containing the proposed and accepted chains. Note that the size of the chains can be tremendously large for real data sets, so the chain should only be saved for small diagnostic simulations.

The user can work with the GUI or write his or her own script to call different functions from the class. The gui displays eight panels. The DATA-panel has buttons to load the raw data and drifts. The RJStruct-panel tabulates default values for the RJMCMC parameters, which can be altered by the user. The P_Burnin-panel and P_Trial-panel let the user change the probabilities of proposing different jumps in the burn-in part of the chain and the trial portion afterwards. The SMA-panel displays the parameters required by the single-emitter code. Some input parameters of the code are included in the Parameters-panel. The Threshold-panel and FrameConnect-panel contain the post-processing parameters. The post-processing can be disabled by unchecking the boxes next to their panels.

processData()

By pressing the Run-button in the gui, the method processData() is called inside a for-loop which loads a data set in every iteration and sends it to the analyzeData() method. At the end, it saves all the output structures and the posterior reconstruction.

analyzeData()

analyzeData() is the main method in the RJ-class from which all the other analysis methods are called. The first function called inside this method is gainCorrection(), which corrects for the gain and offset noise. Next, findPriors() is called to find the intensity priors for the signal and background emitters and also the prior for the offset background. Then, findPSF_SMA() calculates the numerical PSF using the raw data. After that, the sequence of the frames are divided into a number of subsequences equal to the number of parallel workers available on the machine. A parfor-loop is then called and the workers process each subsequence of the frames individually. The user has the option to use a simple for-loop as well. The method makeSubRegions() is called inside the for-loop, dividing the frames into subregions for speed purposes. Next, the subregions are sent to analyzeROIs() to be processed. The subregions overlap to remove artifacts at their edges, so the found emitters in the overlapping area need to be removed. This task is done by the removeOverlapping() method. Then, stitchROIs() stitches together all the subregions in a frame, and then assembles the frames to get the final results for a subsequence. Finally, when the parfor-loop is done, the results are retrieved from the different workers and collected together.

findPriors()

findPriors() runs a fast single-emitter algorithm [19] to find the emitters' intensities and the offset background. It then fits a smoothed kernel density estimator to the found intensities. Because the single-emitter algorithm sometimes fits two or more very close emitters by a single emitter with higher intensity, we might get smaller peaks at higher intensities than the main peak, which is unrealistic. To eliminate those following unrealistic peaks in the returned intensity prior, we calculate the gradient of the prior and use that to remove the spurious smaller peaks described. The resulting distribution is returned as the numerical prior for signal intensities. The background emitters' intensity prior is taken as an exponential distribution with an average equal to the mean of the found intensities divided by a user provided parameter (BgPriorRatio). Moreover, this method returns a gamma distribution fit to the found offset backgrounds.

findPSF_SMA()

findPSF_SMA() calculates the numerical PSF for each subregion. This is important because the PSF might be a function of the position. It makes use of the single-emitter code to fit a portion of the data after which the outputs are filtered to find isolated emitters. Next, the PSF is created by shifting and averaging over more than 100 high signal, found single emitters. A $4\times$ sub-sampled PSF is created by padding the Fourier transform. findPSF_SMA() returns a MATLAB cell containing the numerical PSF model for each subregion.

analyzeROIs()

The subregions and found priors are sent to analyzeROIs() for processing. Inside this function, each subregion is analyzed individually in every iteration of the for-loop described in analyzeData(). First, the rjmcmc() method is called to implement the RJMCMC algorithm for a subregion. It returns the chain which is the input to the findMap() method. Next, findMap() finds the most likely model in the chain and returns the parameters associated with that model. This model is used next to initialize an MCMC chain inside the mcmc() method. MCMC only tries within model moves to find more accurate positions and intensities of the found emitters. Finally, the results from mcmc() plus the posterior image of the subregion are returned as outputs of analyzeROIs().

findMap()

findMap() takes the chain from the rjmcmc() method and uses the histogram of the number of the found emitters to find the most repeated model. It then extracts the states of the chain corresponding to that model and uses the k-means algorithm to find the positions, intensities and their associated errors, which are used later to initialize the MCMC algorithm. The k-means step is necessary because adding and subtracting many emitters from the chain makes it impossible to recognize a particular emitter in different states of the chain. The assembleResults() method is called to put together the results from all the different data sets and performs the post-processing. In the following, a short description of some of the visualization and diagnosis methods are given.

chainAnimation()

chainAnimation() takes the chain and the subregion number plus the true positions (for simulated data). When the user call this function, a gui pops up, which can then be used to look at different states of the accepted chain and the proposed chain, the overlay of the data with the model for the accepted and proposed chains, the emitters' parameters for both chains and also a plot of the emitters' positions. It is a very useful tool and can be employed for either diagnostic purposes or for getting a better grasp of the RJMCMC algorithm.

makeModel()

makeModel() is a method for diagnosis and also for getting better insight into the RJMCMC algorithm. It takes a subregion and its corresponding chain and makes a 3D stack of images, where each slice of the stack is an overlay of the data with a model from a different state of the chain. This is a useful tool to look at the overlay of models produced from different states of the chain with experimental data, where the true positions are not known and the user cannot use the chainAnimation() method.

4 Supplementary Note 4: Numerical PSF interpolation

In the case of bright emitters, such as that from DNA-PAINT data, the 2D Gaussian approximation or theoretical models of the microscope PSF are not adequate for multiple-emitter fitting [28]. In these situations, one can use experimentally acquired PSFs [28–32]. Our approach to estimating the numerical PSF was described in the previous section (findPSF_SMA()). The N subsampled PSF is stored in a $4D$ array where the third dimension contains N samples along the X-axis and the fourth dimension contains N samples along the Y-axis. By linear interpolation along the X and Y axes and scaling by the number of photons, one obtains the PSF with its center at a desired location. This procedure is repeated for all the existing emitters to generate the model.

References

[26] Brooks, S., Gelman, A., Jones, G. L. & Meng, X.-L. Handbook of Markov Chain Monte Carlo. (CRC Press, 2011).

[27] Huang, F. *et al.* Video-rate nanoscopy using sCMOS camera-spesific single-molecule localization algorithms. *Nat Methods* **10**, 653-658 (2013).

[28] Liu, S. Kromann, E. B., Krueger, W. D., Bewersdorf, J. & Lidke, K. A., Three dimensional single molecule localization using a phase retrieved pupil function, *Opt. Express* **21** 29462-29487 (2013).

[29] Quirin, S., Pavani, S. R. P. & Piestun, R., Optimal 3D single-molecule localization for superresolution microscopy with aberrations and engineered point spread functions, *Proc. Natl. Acad. Sci. USA* **109**, 675-679 (2012).

[30] Baddeley, D., Cannell, M. B. & Soeller, C., Three-Dimensional Sub-100 nm Super-Resolution Imaging of Biological Samples Using a Phase Ramp in the Objective Pupil, *Nano Res.* **4**, 589-598 (2011).

[31] Babcock, H. P., & Zhuang, X., Analyzing Single Molecule Localization Microscopy Data Using Cubic Splines, *Sci. Rep.* **7**, 552 (2017).

[32] Li, Y., Mund, M., Hoess, P., Deschamps, J., Matti, U., Nijmeijer, B., Sabinina, V. J., Ellenberg, J., Schoen, I., & Ries, J., Real-time 3D singlemolecule localization using experimental point spread functions, *Nat Methods* **15**, 367-369 (2018).
