# Supplementary figures and images for: Bayesian Multiple Emitter Fitting using Reversible Jump Markov Chain Monte Carlo
Source: Sci Rep. 2019 Sep 24;9:13791. doi: 10.1038/s41598-019-50232-x (PMC6760159; doi:10.1038/s41598-019-50232-x)

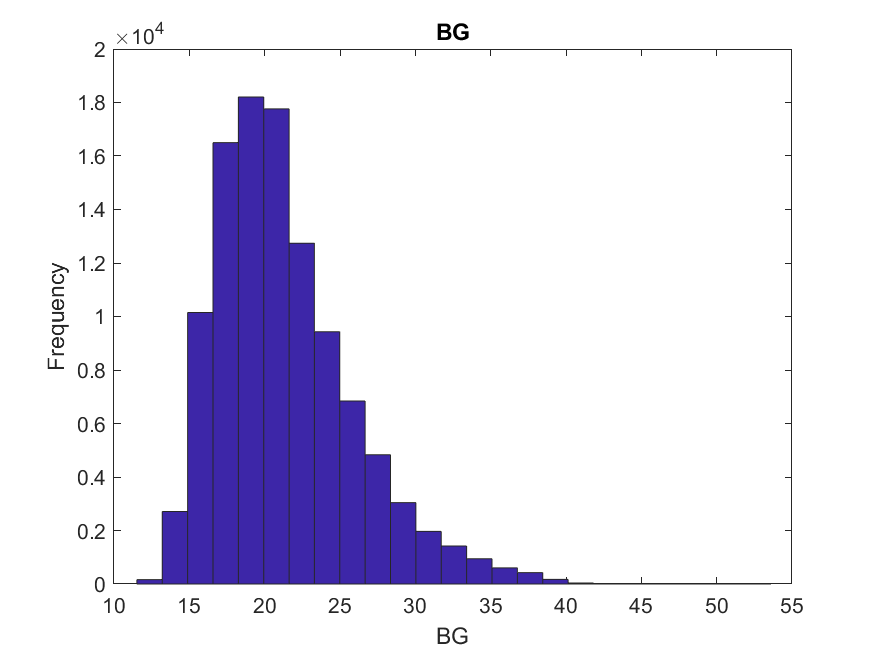

Supplement: Supplementary file 2 — The algorithm package [file 41598_2019_50232_MOESM2_ESM.zip › BAMF/Examples/Actin/BG.png]

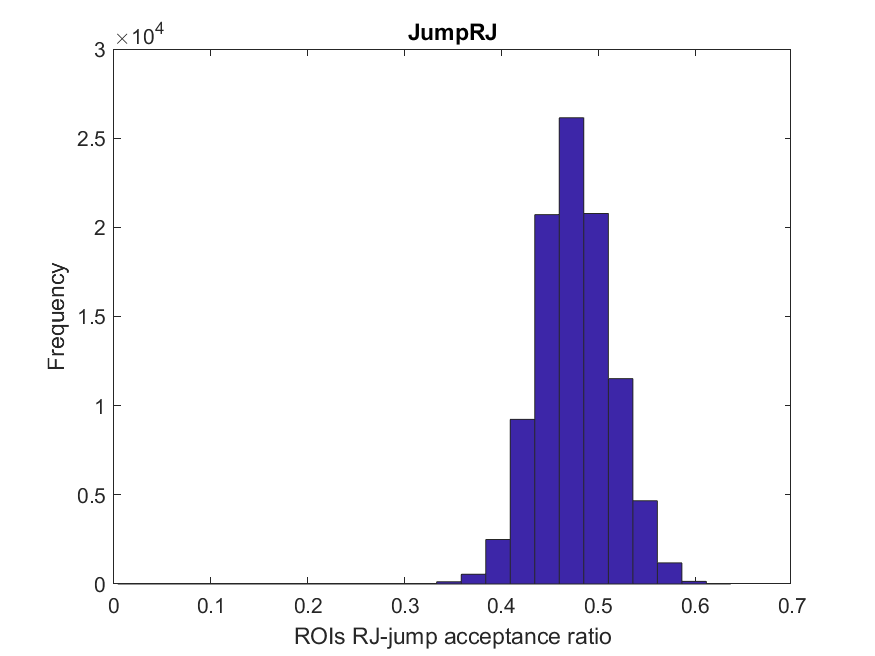

Supplement: Supplementary file 2 — The algorithm package [file 41598_2019_50232_MOESM2_ESM.zip › BAMF/Examples/Actin/JumpRJ.png]

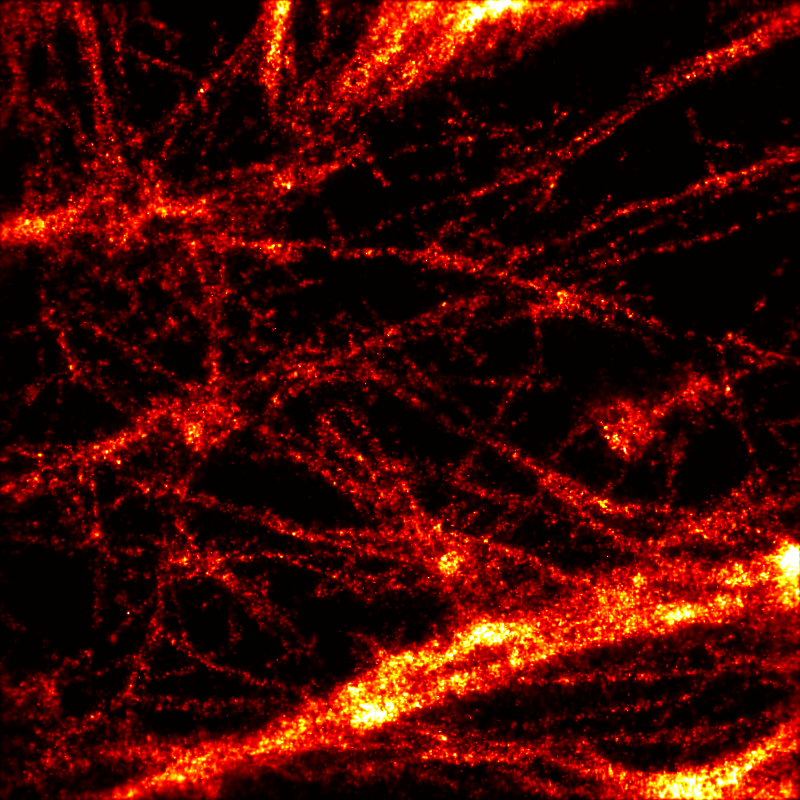

Supplement: Supplementary file 2 — The algorithm package [file 41598_2019_50232_MOESM2_ESM.zip › BAMF/Examples/Actin/MCMC.png]

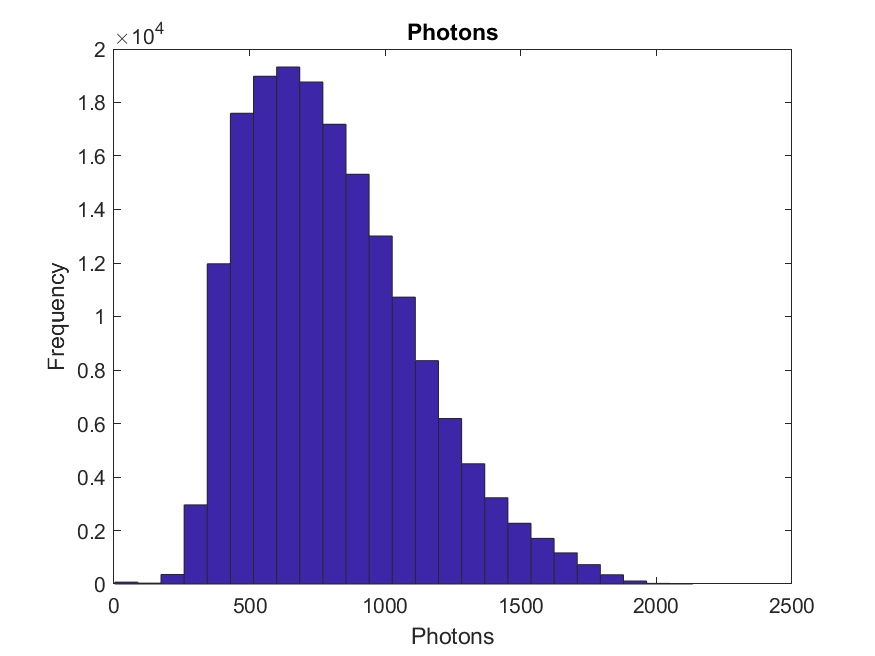

Supplement: Supplementary file 2 — The algorithm package [file 41598_2019_50232_MOESM2_ESM.zip › BAMF/Examples/Actin/Photons.png]

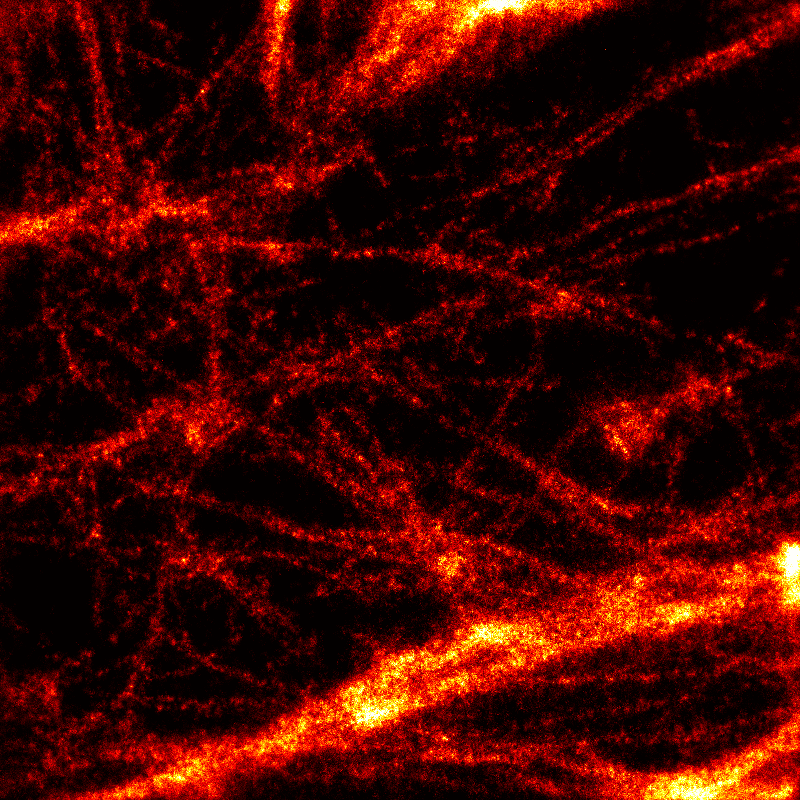

Supplement: Supplementary file 2 — The algorithm package [file 41598_2019_50232_MOESM2_ESM.zip › BAMF/Examples/Actin/Posterior.png]

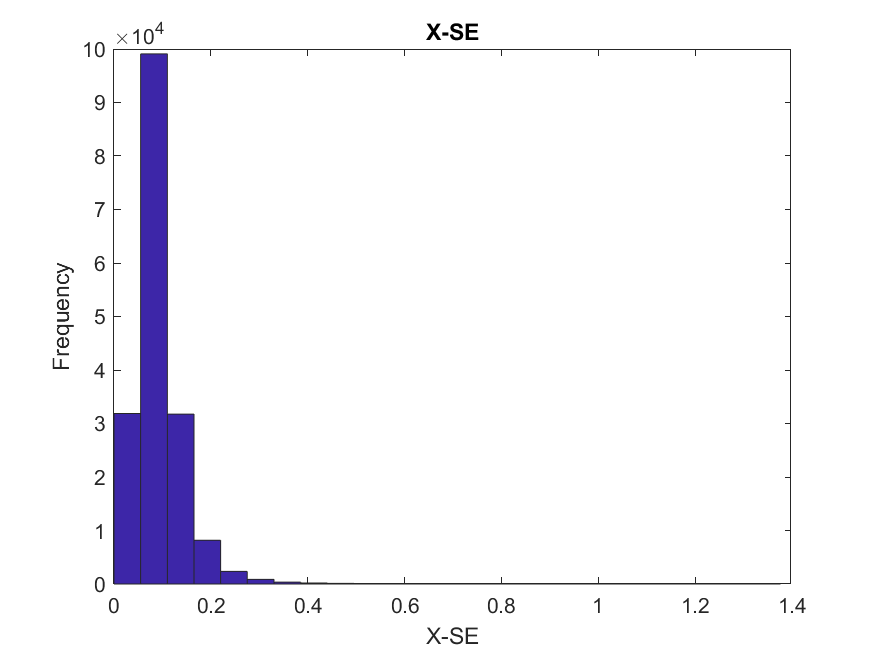

Supplement: Supplementary file 2 — The algorithm package [file 41598_2019_50232_MOESM2_ESM.zip › BAMF/Examples/Actin/X-SE.png]

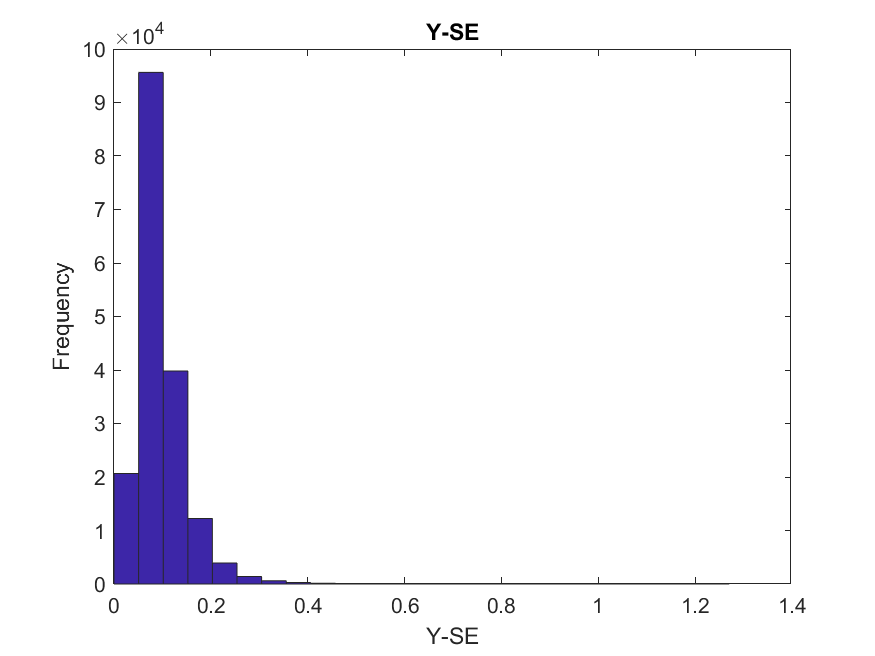

Supplement: Supplementary file 2 — The algorithm package [file 41598_2019_50232_MOESM2_ESM.zip › BAMF/Examples/Actin/Y-SE.png]

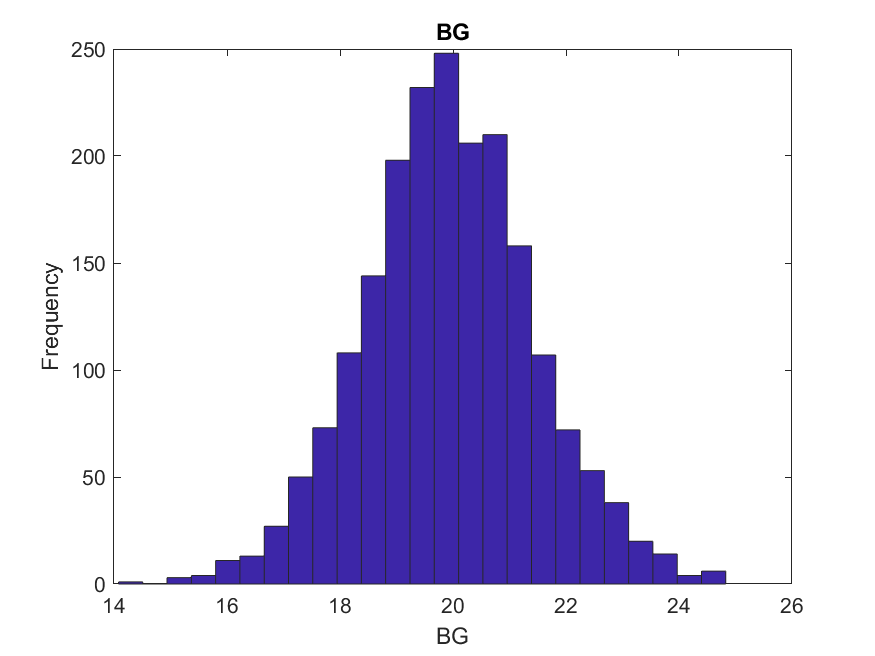

Supplement: Supplementary file 2 — The algorithm package [file 41598_2019_50232_MOESM2_ESM.zip › BAMF/Examples/Circle/BG.png]

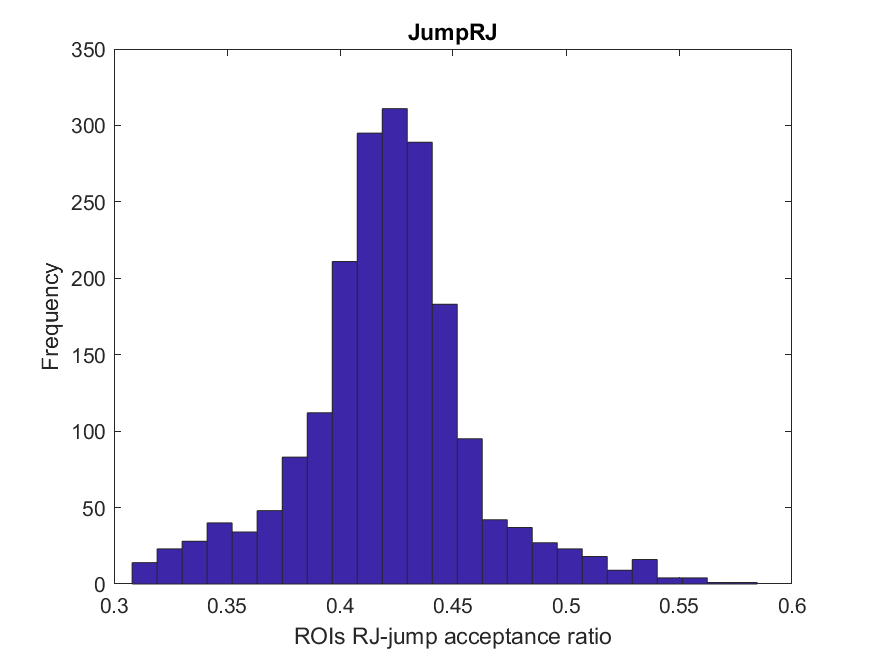

Supplement: Supplementary file 2 — The algorithm package [file 41598_2019_50232_MOESM2_ESM.zip › BAMF/Examples/Circle/JumpRJ.png]

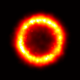

Supplement: Supplementary file 2 — The algorithm package [file 41598_2019_50232_MOESM2_ESM.zip › BAMF/Examples/Circle/MCMC.png]

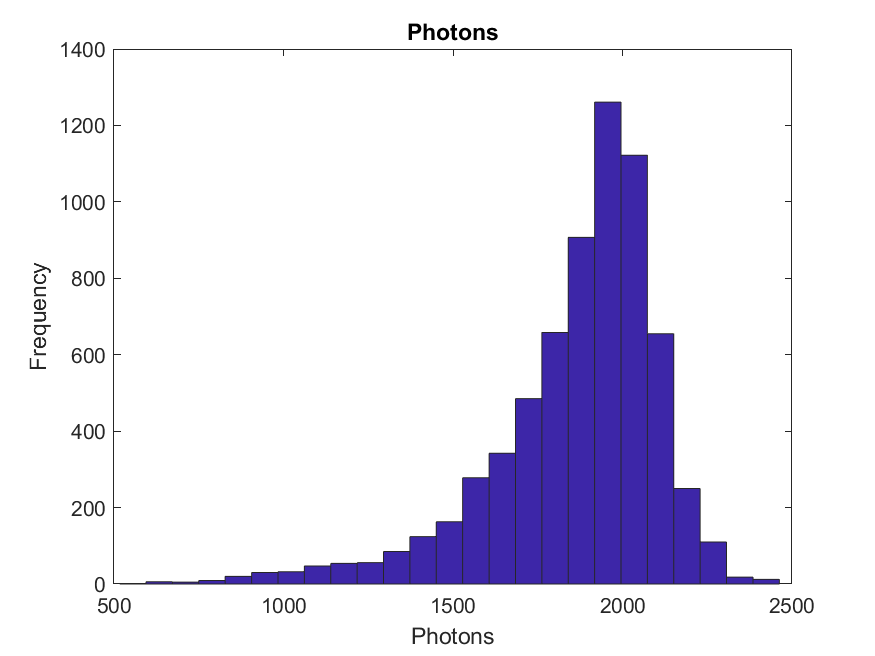

Supplement: Supplementary file 2 — The algorithm package [file 41598_2019_50232_MOESM2_ESM.zip › BAMF/Examples/Circle/Photons.png]

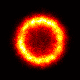

Supplement: Supplementary file 2 — The algorithm package [file 41598_2019_50232_MOESM2_ESM.zip › BAMF/Examples/Circle/Posterior.png]

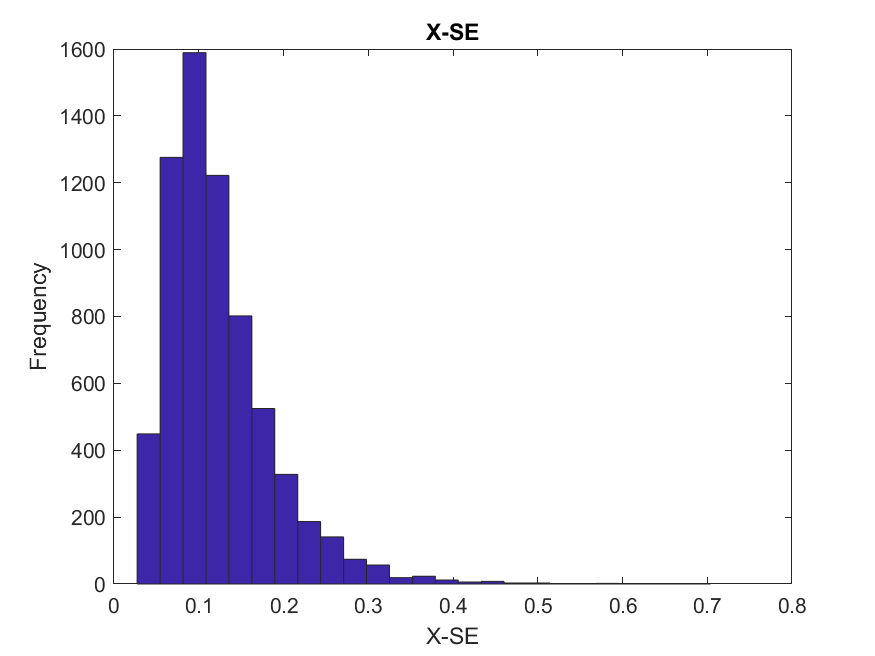

Supplement: Supplementary file 2 — The algorithm package [file 41598_2019_50232_MOESM2_ESM.zip › BAMF/Examples/Circle/X-SE.png]

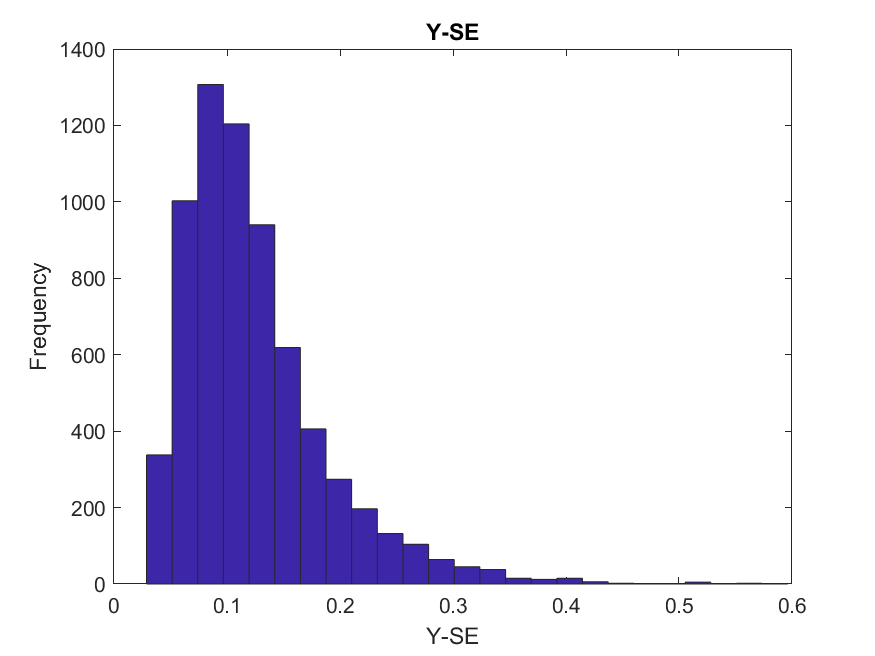

Supplement: Supplementary file 2 — The algorithm package [file 41598_2019_50232_MOESM2_ESM.zip › BAMF/Examples/Circle/Y-SE.png]
